# Gold-catalyzed intermolecular coupling of sulfonylacetylene with allyl ethers: [3,3]- and [1,3]-rearrangements

**DOI:** 10.3762/bjoc.9.198

**Published:** 2013-08-22

**Authors:** Jungho Jun, Hyu-Suk Yeom, Jun-Hyun An, Seunghoon Shin

**Affiliations:** 1 Department of Chemistry and Institute for Natural Sciences, Hanyang University, Seoul, 133-791, Korea

**Keywords:** gold catalysis, intermolecular coupling, [1,3]-rearrangement, [3,3]-sigmatropic rearrangement, sulfonylacetylene

## Abstract

Gold-catalyzed intermolecular couplings of sulfonylacetylenes with allyl ethers are reported. A cooperative polarization of alkynes both by a gold catalyst and a sulfonyl substituent resulted in an efficient intermolecular tandem carboalkoxylation. Reactions of linear allyl ethers are consistent with the [3,3]-sigmatropic rearrangement mechanism, while those of branched allyl ethers provided [3,3]- and [1,3]-rearrangement products through the formation of a tight ion–dipole pair.

## Introduction

Homogeneous gold catalysis has been established during the last decade as a prominent tool in organic chemistry, mediating a variety of C–C and C–X (heteroatom) bond formations, various tandem reactions and rearrangements [1]. Despite these significant advances, overcoming entropic penalty in intermolecular coupling of alkenes with alkynes is still a major challenge in gold catalysis, reflected by the scarcity of such examples [2–5]. Earlier examples in this vein include intermolecular reactions of electron-rich arenes and heteroarenes [2–3]. More recently, relatively polarized 1,1-disubstituted olefins were also found to react intermolecularly with phenylacetylenes or propiolic acids [4–5].

**Figure 1:**
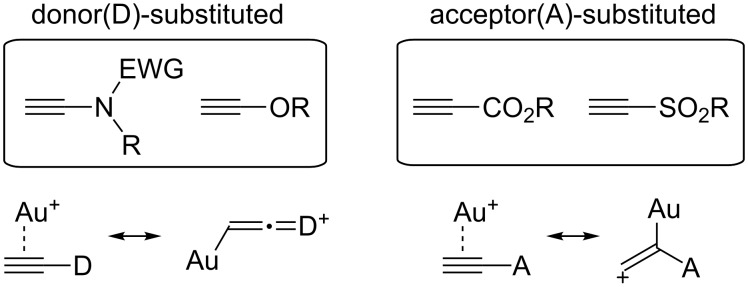
Donor- and acceptor-substituted alkynes for Au-catalyzed intermolecular reactions.

Recently, strategies capitalizing upon donor- or acceptor-polarized alkynes have been introduced, perhaps to enhance the charge interaction and thus to facilitate the intermolecular reactivity (Figure 1). For example, Liu and co-workers have utilized ynamides for intermolecular [4 + 2] and [2 + 2 + 2] reactions with alkenes [6]. On the other hand, Shin and co-workers have adopted propiolic acids and alkynyl sulfones for formal enyne cross metathesis (*f-*EYCM) [5]. These examples allow for an effective alkyne–alkene coupling under mild reaction conditions (rt) with as little as 1.5 ~ 2 equiv of an excess component.

Expanding upon the intermolecular coupling reactions of readily available alkenes with alkynes would significantly enhance the synthetic utility of gold catalysis and therefore should find fruitful applications. While it has been known for a long time that allyl alcohols undergo intermolecular alkoxylation-[3,3]-sigmatropic rearrangement under Ag(I) or Au(I) catalysis [7–8], allyl ethers that are less nucleophilic due to steric reasons react more slowly and have not been known to undergo similar reactions until recently. In our previous work [9], it was shown that ester-substituted alkynes underwent an efficient intermolecular carboalkoxylation with allyl ethers via a tandem conjugate addition and a [3,3]-sigmatropic rearrangement [10–12]. Preliminary results in the above studies [5,9] have demonstrated that a polarizing effect of the sulfonyl substituent on the alkyne is highly effective in promoting the reaction under a mild condition with relatively low amount of excess reactants. We report herein the details of our investigation on the intermolecular reactions of alkynyl sulfones with allyl ethers aimed at definition of the substrate scope and at elucidation of the competitive [1,3], and [3,3]-rearrangement pathways and their respective mechanisms.

## Results and Discussion

At the outset, the effect of ligand, counter-anion and solvent in the Au-catalyzed coupling of *p-*toluenesulfonylacetylene (**1**) with an allyl ether **2** was examined (Table 1). When Au(L)SbF_6_ (L = di-*t-*butyl-*o-*biphenylphosphine, JohnPhos) formed in situ was used as catalyst, the reaction was more efficient in chlorinated solvents rather than polar aprotic or aromatic hydrocarbon solvents (Table 1, entries 1–7). Contrary to the previous [4 + 2] cycloaddition, formal enyne cross metathesis or [2 + 2] cycloaddition [4–5] where JohnPhos ligand showed the best performance, the optimal ligand for the current carboalkoxylation was different. While the role of electron density of the ligand was less obvious, the steric bulk on the ligand clearly seemed to retard the reaction and a less bulky PPh_3_ was chosen as the optimal ligand (Table 1, entries 8–13). Further optimization with regard to reactants stoichiometry was conducted. An increased rate was observed when the amount of allyl ethers increased up to 3 equivalents. However, an increase in the amount of sulfonylacetylene (**1**) was less effective (Table 1, entries 14–18). Finally, SbF_6_^–^ turned out to be an optimal counter-anion for cationic [Au(PPh_3_)]^+^ (Table 1, entries 19–21). A control experiment with AgSbF_6_ as the only catalyst led to no reaction (Table 1, entry 22). Apparently, unlike allyl alcohols, sterically bulkier allyl ethers do not undergo O*-*attack on the alkyne in the presence of Ag-catalyst [7].

**Table 1:** Optimization of the reaction conditions.^a^

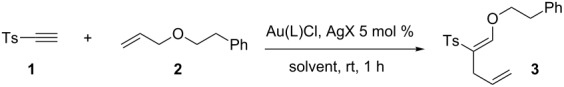					

Entry	Ligand	AgX	**1**:**2**	Solvent	Yield^b^

1	JohnPhos	AgSbF_6_	1:1.2	CHCl_3_	32
2	JohnPhos	AgSbF_6_	1:1.2	DCM	30
3	JohnPhos	AgSbF_6_	1:1.2	DCE	23
4	JohnPhos	AgSbF_6_	1:1.2	CH_3_NO_2_	20
5	JohnPhos	AgSbF_6_	1:1.2	CH_3_CN	0
6	JohnPhos	AgSbF_6_	1:1.2	THF	0
7	JohnPhos	AgSbF_6_	1:1.2	PhH	3
8	P(OC_6_H_5_)_3_	AgSbF_6_	1:1.2	CHCl_3_	36
9	PPh_3_	AgSbF_6_	1:1.2	CHCl_3_	48
10	IMes^c^	AgSbF_6_	1:1.2	CHCl_3_	22
11	IPr^d^	AgSbF_6_	1:1.2	CHCl_3_	19
12	P(C_6_F_5_)_3_	AgSbF_6_	1:1.2	CHCl_3_	10
13	PtBu_3_	AgSbF_6_	1:1.2	CHCl_3_	7
14	PPh_3_	AgSbF_6_	1:1	CHCl_3_	40
15	PPh_3_	AgSbF_6_	1:2	CHCl_3_	68
16	PPh_3_	AgSbF_6_	1:3	CHCl_3_	77
17	PPh_3_	AgSbF_6_	1:5	CHCl_3_	74
18	PPh_3_	AgSbF_6_	5:1	CHCl_3_	53
19	PPh_3_	AgOTf	1:3	CHCl_3_	48
20	PPh_3_	AgNTf_2_	1:3	CHCl_3_	44
21	PPh_3_	AgBF_4_	1:3	CHCl_3_	22
22	–^e^	AgSbF_6_	1:3	CHCl_3_	0

^a^Conditions: in situ formed catalyst from Au(L)Cl and AgX (5 mol % each); rt, 1 h. ^b^Crude yield based on the internal reference (*N,N-*dimethylacetamide). ^c^IMes = 1,3-dimesitylimidazol-2-ylidene. ^d^IPr = 1,3-bis(2,6-diisopropylphenyl)imidazol-2-ylidene. ^e^In the absence of Au(L)Cl.

**Scheme 1:**
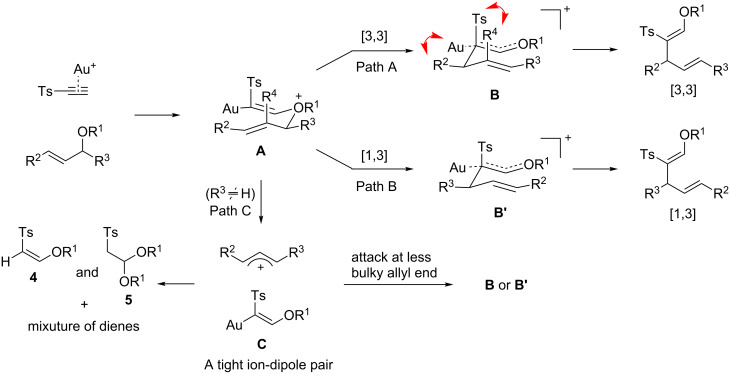
Proposed mechanism of the [3,3]- and [1,3]-rearrangement.

With the above optimized conditions in hand, the scope of the carboalkoxylation of sulfonylacetylene was examined (Table 2). The alkoxy group in the ethers **2** had an impact on the efficiency of the current tandem carboalkoxylation. The reaction of methyl ether **2a** was accompanied by a side product **4** (R^1^ = Me) resulting from a premature dissociation of the allyl cation fragment before the rearrangement, decreasing the yield of desired **3a** (Table 2, entry 1). However, **2b** having sterically bulky secondary (IPr) or primary alkoxy groups underwent smooth reactions (Table 2, entries 2 and 3). It is reasonable to assume that a bulky group R^1^ would decelerate the initial O*-*attack on the alkyne. However, once the Au-bound oxonium ion (**A** in Scheme 1) is formed, the resulting rearrangement seems to be facilitated by the presence of a bulky substituent at R^1^.

**Table 2:** Scope of the carboalkoxylation of sulfonyl acetylene (**1**).^a^

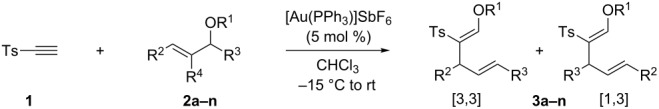							

Entry	R^1^	R^2^	R^3^	R^4^	Product	Yield^b^ (%)	[3,3]/[1,3]

1	Me	*n-Pr*	H	H	**3a**	53	9:1
2	IPr	*n-Pr*	H	H	**3b**	67	10:1
3	(CH_2_)_2_Ph	*n-Pr*	H	H	**3c**	72	8:1
4	(CH_2_)_2_Ph	Cy	H	H	**3d**	54	4:1
5^c^	(CH_2_)_2_Ph	(CH_2_)_2_Ph	H	H	**3e**	75	13:1
6	(CH_2_)_2_Ph	Me	H	H	**3f**	75	14:1
7^c^	(CH_2_)_2_Ph	H	H	H	**3g**	74	–
8	Allyl	*n-Pr*	H	H	**3h**	60	8:1
9^c^	Me	*n-Pr*	Me	H	**3i**	40	1:1.7^d^
10	(CH_2_)_2_Ph	(CH_2_)_3_		H	**3j**	23	–^e^
11	nC_8_H_17_	*n-Pr*	Me	H	**3k**	31	1:1.4^f^
12	nC_8_H_17_	H	Me	H	**3l**	34	>20:1^g^
13	(CH_2_)_2_Ph	Me	Me	H	**3m**	18	–
14	(CH_2_)_2_Ph	H	H	Me	**3n**	23	–

^a^Conditions: Allyl ether (3.0 equiv) and **1** (1 equiv) in the presence of in situ formed [Au(PPh_3_)]SbF_6_ (5 mol %) in CHCl_3_ from −15 °C to rt, 2.5 h. ^b^Isolated yield after chromatography. ^c^Characterization data have been previously provided ([9]). ^d^15% of **4** (R^1^ = Me) was observed for the reaction of **3i**. ^e^10% of **4** and 43% of **5** (R^1^ = (CH_2_)_2_Ph) was observed for the reaction of **3j**. ^f^20% of **4** (R^1^ = *n*-C_8_H_17_) was observed for the reaction of **3k**. ^g^3% of **4** (R^1^ = *n*-C_8_H_17_) was observed for the reaction of **3i**.

The substituents on the allyl unit also affected the reaction significantly. A cyclohexyl group as γ-substituent (R^2^) led to a slower reaction, delivering **3d** only in 54% yield, with a concomitant decrease in the ratio of [3,3]- versus [1,3]-rearrangement products, while primary alkyl groups as R^2^ were well accommodated (Table 2, entries 4–6). These indicated that a steric crowding in the proposed [3,3]-sigmatropic rearrangement transition state (Path A in Scheme 1) resulted in a sluggish reaction, but affected the competitive [1,3]-rearrangement less severely. It is noteworthy that an unsubstituted (R^2^, R^3^ = H) allyl ether **2g** afforded **3g** in a good yield (Table 2, entry 7), unlike the reactions with propiolates [9] where only ~21% of carboalkoxylation product was obtained. However, a competition experiment using **3h** having two different allyl groups showed that the more electron-rich allyl unit migrated exclusively (Table 2, entry 8), clearly indicating that an electron-rich R^2^ substituent accelerated the [3,3]-sigmatropic rearrangement. In the presence of R^3^ (α-)substituent (**2i–k**), however, both the rate and the yield of the reaction was significantly compromised and the reaction was accompanied by the extensive formation of either **4** or **5** (Table 2, entries 9–13). It is interesting to note that the ratio of [3,3]- versus [1,3]-rearrangement products reversed dramatically in these cases in favor of [1,3]-rearrangement (Table 2, entries 9 and 11), most probably because of a facile ionization of the C–O bond leading to an allyl cation and **C** (Path C, Scheme 1). Intriguingly, **2l** having a α-Me substituent and no γ-substituent provided an exclusive formation of an apparent [3,3]-rearrangement product **3l** (Table 2, entry 11). These experiments indicated that for those having an α-substituent, the steric nature of R^2^ and R^3^ substituents determined the ratio of [3,3]- versus [1,3]-products. Finally, R^4^ substituent at the allyl group (**2n**) retarded the transformation severely, indicating an unfavorable steric interaction. Unlike previous intramolecular [3,3]-sigmatropic rearrangements [13–14], the γ,γ-disubstituted allyl ethers derived from geraniol or nerol were completely inactive, most probably due to steric reasons as in the case of **2d**.

The proposed mechanism accounting for the above reactivity profile is depicted in Scheme 1. Having a stronger acceptor (Ts), **1** requires less amount of an excess reactant for the formation of the key intermediate **A** than propiolates and allows for the migration of even less electron-rich allyl group as in **2g** [9]. A key mechanistic difference is the facile cleavage of the allyl C–O bond in **A** induced by the stronger polarizing effect of the tosyl group to give **C** and allyl cation, which evolves into **4** and a mixture of dienes. This was especially severe for substrates having an α-substituent (**2i–l**) where the stability of the resulting allyl cation further facilitates the ionization. The combination of the resulting intermediate **C** and the allyl cation occurred at the sterically less hindered allyl end, leading to preferential formation of the [1,3]-rearrangement product for **2i** and **2k** and an apparent [3,3]-rearrangement product for **2l**. For those without α-substituents, the negative influence of steric bulk at the R^2^ (**2d**) and R^4^ (**2n**) indicates a compact transition state in the concerted [3,3]-sigmatropic rearrangement (Path A) where repulsion between R^2^ and Au(L) and between R^4^ and Ts decelerates the reaction, respectively.

**Scheme 2:**
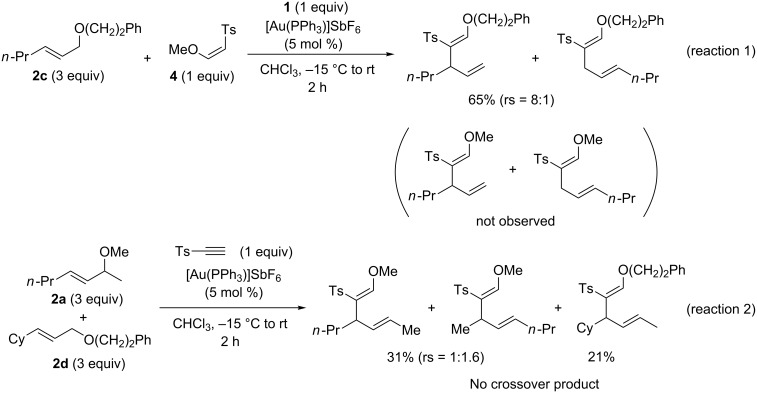
Experiments to investigate the reaction mechanism.

To examine the possible role of **4** in the carboalkoxylation, **4** (R^1^ = Me) was added in a reaction mixture of **2c** in the presence of the Au-catalyst. No product resulting from a combination of **4** with the allyl fragment in **2c** was observed (Scheme 2, reaction 1), eliminating the role of **4** as the nucleophilic component along the Path C to **B/B’**. Furthermore, in a cross-over experiment with an equimolar mixture of **2a** and **2d** in the presence of the Au-catalyst, no cross-over product was observed by GC–MS and NMR spectrometry, indicating the [3,3]- and [1,3]-rearrangement occurred intramolecularly (Scheme 2, reaction 2). This was further confirmed by the cross-over experiment employing **2i** and **2m**, two α-substituted allyl ethers [9]. The absence of cross-over in the latter experiment strongly indicated that the formation of a tight ion-dipole pair between **C** and the allyl cation in the reactions of α-substituted allyl ethers (Path C). A concerted [1,3]-sigmatropic rearrangement (Path B) seems less likely because such a rearrangement should occur through *antara*-facial selectivity due to the orbital symmetry.

## Conclusion

Gold catalyzed intermolecular coupling of allyl ethers with sulfonylacetylene has been reported. The strong polarizing effect of the sulfonyl group induced an effective intermolecular tandem carboalkoxylation with a lower amount of the excess reactant. However, it is accompanied by a significant amount of byproduct(s) such as **4** and **5**, resulting from the dissociation of the allyl C–O cleavage. While the linear allyl ethers preferred [3,3]-sigmatropic rearrangements, the presence of an α-substituent led to a facile dissociation of the allyl C–O bond leading to [1,3]- or [3,3]-rearrangement products depending on the substituents. For both [3,3]- and [1,3]-rearrangements, control experiments confirmed the intramolecular mechanism of the allyl migration. Our current efforts are aimed at the elucidation of the exact nature of the [1,3]-rearrangement pathway with its stereochemical consequences and at the synthetic applications of the resulting products.

## Supporting Information

File 1 Characterization of starting materials, general procedure for the carboalkoxylation, characterization of products, and ^1^H and ^13^C NMR spectra of all new compounds.
