# Supplementary material for: Gold-catalyzed intermolecular coupling of sulfonylacetylene with allyl ethers: [3,3]- and [1,3]-rearrangements
Source: Beilstein J Org Chem. 2013 Aug 22;9:1724–9. doi: 10.3762/bjoc.9.198 (PMC3778381; doi:10.3762/bjoc.9.198)
Supplement: File 1 — Characterization of starting materials, general procedure for the carboalkoxylation, characterization of products, and 1H and 13C NMR spectra of all new compounds. [file Beilstein_J_Org_Chem-09-1724-s001.pdf]

Supporting Information

for

Gold-catalyzed intermolecular coupling of sulfonylacetylene with allyl ethers: [3,3]- and [1,3]-rearrangements

*Jungho Jun, Hyu-Suk Yeom, Jun-Hyun An and Seunghoon Shin**

Address: Department of Chemistry and Institute for Natural Sciences, Hanyang University, Seoul, 133-791, Korea

Email: Seunghoon Shin - sshin@hanyang.ac.kr

*Corresponding author

Characterization of starting materials, general procedure for the carboalkoxylation, characterization of products, and ^{1}H and ^{13}C NMR spectra of all new compounds

1. Characterization of starting materials
2. General procedure for the carboalkoxylation
3. Characterization of products
4. Scanned copies of the spectra for substrates
5. Scanned copies of the spectra for products

1. Characterization of starting materials

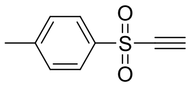

1

^{1}H NMR ($CDCl_3$, 400 MHz): δ 7.90 (d, *J* = 8.6 Hz, 2H), 7.40 (d, *J* = 8.2 Hz, 2H), 3.47 (s, H), 2.48 (s, 3H); ^{13}C NMR ($CDCl_3$, 100 MHz): δ 146.1, 138.0, 130.2, 127.8, 81.2, 80.5, 21.9.

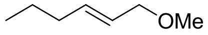

2a

^{1}H NMR ($CDCl_3$, 400 MHz): δ 5.70 (dt, *J* = 15.6, 7.7 Hz, H), 5.55 (dt, *J* = 15.2, 6.2 Hz, H), 3.86 (d, *J* = 6.2 Hz, 2H), 3.32 (s, 3H), 2.03 (q, *J* = 7.0 Hz, 2H), 1.41 (sextet, *J* = 7.4 Hz, 2H), 0.90 (t, *J* = 7.0 Hz, 3H); ^{13}C NMR ($CDCl_3$, 100 MHz): δ 134.8, 126.3, 73.4, 57.7, 34.5, 22.3, 13.8.

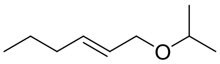

2b

^{1}H NMR ($CDCl_3$, 400 MHz): δ 5.68 (dt, *J* = 15.2, 6.7 Hz, H), 5.56 (dt, *J* = 15.7, 5.8 Hz, H), 3.91 (d, *J* = 5.9, 2H), 3.61 (septet, *J* = 6.2 Hz, H), 2.02 (q, *J* = 7.0 Hz, 2H), 1.40 (sextet, *J* = 7.4 Hz, 2H), 1.16 (d, *J* = 5.8, 6H), 0.90 (t, *J* = 7.0 Hz, 3H); ^{13}C NMR ($CDCl_3$, 100 MHz): δ 134.0, 127.2, 70.7, 69.1, 34.5, 22.4, 22.3, 13.9.

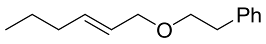

2c

^{1}H NMR ($CDCl_3$, 400 MHz): δ 7.32-7.19 (m, 5H), 5.69 (dt, *J* = 15.3, 6.6 Hz, H), 5.55 (dt, *J* = 15.2, 6.3 Hz, H), 3.95 (d, *J* = 6.2 Hz, 2H), 3.64 (t, *J* = 7.4 Hz, 2H), 2.91 (t, *J* = 7.4 Hz, 2H), 2.03 (q, *J* = 7.0 Hz, 2H), 1.41 (sextet, *J* = 7.5 Hz, 2H), 0.91 (t, *J* = 7.4 Hz, 3H); ^{13}C NMR ($CDCl_3$, 100 MHz): δ 139.1, 134.6, 129.0, 128.5, 126.6, 126.3, 71.8, 71.1, 36.5, 34.5, 22.4, 13.8.

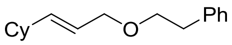

2d

^{1}H NMR ($CDCl_3$, 400 MHz): δ 7.40-7.16 (m, 5H), 5.63 (dd, *J* = 15.7, 6.3 Hz, H), 5.49 (dt, *J* = 15.6, 6.2 Hz, H), 3.93 (d, *J* = 5.8, 2H), 3.62 (t, *J* =7.4 Hz, 2H), 2.90 (t, *J* = 7.4 Hz, 2H), 2.08-1.88 (m, H), 1.82-1.59 (m, 5H); ^{13}C NMR ($CDCl_3$, 100 MHz): δ 140.4, 139.1, 129.0, 128.5, 126.3, 123.8, 72.0, 71.1, 40.5, 36.5, 32.9, 26.3, 26.2.

(E/Z = 16:1)

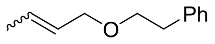

2f

^{1}H NMR ($CDCl_3$, 400 MHz): δ 7.40-7.32 (m, E&Z isomer 5H), 5.70 (dq, *J* = 15.2, 6.6 Hz, E isomer H), 5.57 (dtd, *J* = 15.3, 6.3, 1.2 Hz, E isomer H), 4.05 (d, *J* = 6.7 Hz, Z isomer 2H), 3.91 (d, *J* = 6.2 Hz, E isomer 2H), 3.62 (t, *J* = 7.4 Hz, E isomer 2H), 2.90 (t, *J* = 7.5 Hz, E isomer 2H), 1.71 (d, *J* = 6.2 Hz, E isomer 3H), 1.62 (d, *J* = 6.7 Hz, Z isomer 3H); ^{13}C NMR ($CDCl_3$, 100 MHz): δ 139.0, 129.4, 128.9, 128.3, 127.6, 126.2, 71.6, 71.0, 36.4, 17.8.

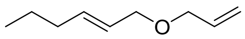

2h

^{1}H NMR ($CDCl_3$, 400 MHz): δ 5.92 (ddt, *J* = 16.0, 10.6, 5.9 Hz, H), 5.70 (dt, *J* = 15.2, 6.3 Hz, H), 5.56 (dt, *J* = 15.2, 6.3 Hz, H), 5.27 (dd, *J* = 17.2, 1.6 Hz, H), 5.18 (dd, *J* = 10.6, 1.2 Hz, H), 3.97 (d, *J* = 5.8 Hz, 2H), 3.93 (d, *J* = 6.3 Hz, 2H), 2.03 (q, *J* = 7.0 Hz, 2H), 1.41 (sextet, *J* = 7.4 Hz, 2H), 0.90 (t, *J* = 7.0 Hz, 3H); ^{13}C NMR ($CDCl_3$, 100 MHz): δ 135.0, 134.9, 126.4, 117.1, 71.1, 71.0, 34.5, 22.4, 13.9.

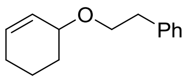

2j

^{1}H NMR ($CDCl_3$, 400 MHz): δ 7.32-7.17 (m, 5H), 5.88-5.81 (m, H), 5.76 (dd, *J* = 2.3, 10.1 Hz, H), 3.85 (s br, H), 3.76-3.60 (m, 2H), 2.90 (t, *J* = 7.4 Hz, 2H),2.10-1.88 (m, 2H), 1.88-1.48 (m, 4H); ^{13}C NMR ($CDCl_3$, 100 MHz): δ 139.2, 130.9, 129.0, 128.4, 127.9, 126.2, 73.1, 69.4, 36.9, 28.4, 25.3, 19.3.

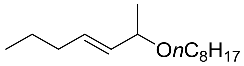

2k

^{1}H NMR ($CDCl_3$, 400 MHz): δ 5.56 (td, *J* = 6.6, 15.6 Hz, H), 5.32 (dd, *J* = 7.8, 15.6 Hz, H), 3.80-3.70 (m, H), 3.48-3.36 (m, H), 3.36-3.18 (m, H), 2.00 (app q, *J* = 7.0 Hz, 2H), 1.60-1.49 (m, 2H), 1.41 (sextet, *J* = 7.4 Hz, 2H), 1.39-1.18 (m, 10H), 1.21 (d, *J* = 6.3 Hz, 3H), 0.90 (t, *J* = 7.4 Hz, 3H), 0.88 (t, *J* = 7.1 Hz, 3H); ^{13}C NMR ($CDCl_3$, 100 MHz): δ 132.6, 132.4, 76.6, 68.2, 34.4, 32.0, 30.1, 29.6, 29.4, 26.4, 22.8, 22.5, 21.9, 14.2, 13.8.

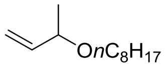

2l

^{1}H NMR ($CDCl_3$, 400 MHz): δ 5.74 (ddd, *J* = 7.0. 10.2, 17.2 Hz, H), 5.16 (d, *J* = 17.2 Hz, H), 5.11 (d, *J* = 10.6 Hz, H), 3.84-3.73 (m, H), 3.49-3.85 (m, H), 3.84-3.72 (m, H), 1.60-1.50 (m, 2H), 1.38-1.20 (m, 10H), 1.23 (d, *J* = 6.7 Hz, 3H), 0.88 (t, *J* = 6.2 Hz, 3H); ^{13}C NMR ($CDCl_3$, 100 MHz): δ 140.9, 115.5, 76.9, 68.6, 32.0, 30.1, 29.6, 29.4, 26.4, 22.8, 21.5, 14.2.

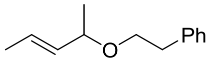

2m

^{1}H NMR ($CDCl_3$, 400 MHz): δ 7.35-7.13 (m, 5H), 5.64-5.52 (m, H), 5.40-5.28 (m, H), 3.83-3.73 (m, H), 3.69-3.57 (m, H), 3.51-3.42 (m, H), 2.94-2.80 (m, 2H), 1.68 (d, *J* = 6.3 Hz, 3H), 1.22 (d, *J* = 6.3 Hz, 3H); ^{13}C NMR ($CDCl_3$, 100 MHz): δ 139.2, 133.5, 129.1, 128.4, 127.5, 126.2, 76.7, 69.1, 36.7, 21.6, 17.8.

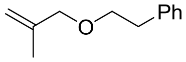

2n

^{1}H NMR ($CDCl_3$, 400 MHz): δ 7.35-7.14 (m, 5H), 4.93 (s, 1H), 4.87 (s, 1H), 3.89 (s, 2H), 3.62 (t, *J* = 7.1 Hz, 2H), 1.70 (s, 3H); ^{13}C NMR ($CDCl_3$, 100 MHz): δ 142.5, 139.2, 129.1, 128.4, 126.3, 112.1, 75.0, 71.1, 36.5, 19.6.

2. General procedure for the carboalkoxylation

A flame-dried test tube was charged with $Au(PPh_3)Cl$ (2.5 mg, 0.005 mmol) and $AgSbF_6$ (1.7 mg, 0.005 mmol). The content was dissolved in anhydrous $CHCl_3$ (1 mL), and allyl ether **2c** (61.3 mg, 0.30 mmol) was added to the mixture. After cooling the mixture to −15 °C, *p*-toluenesulfonylacetylene **1** (18.0 mg, 0.10 mmol) was added in one-lot. The reaction mixture was allowed to warm slowly to ca. 15 °C over 2 h. After the reaction was complete by TLC analysis, the solvent was evaporated and the residue was purified by chromatography (EtOAc:Hex = 1:10) to give 27.7 mg (72%, as a mixture of [3,3]- and [1,3]-product (8:1)) of **3c** as a colorless liquid.

3. Characterization of products

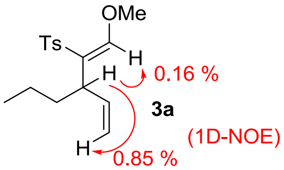

3a

^{1}H NMR ($CDCl_3$, 400 MHz): δ 7.81 (d, *J* = 8.2 Hz, 2H), 7.27 (d, *J* = 7.8 Hz, 2H), 6.23 (s, H), 5.74 (ddd, *J* = 17.2, 9.8, 7.4 Hz), 5.10 (d, *J* = 18.4 Hz, H), 5.06 (d, *J* = 10.9 Hz, H), 3.68 (s, 3H), 3.37 (q, *J* = 7.0 Hz, H), 2.42 (s, 3H), 1.66-1.49 (m, 2H), 1.42-1.30 (m, 2H), 0.91 (t, *J* = 7.4 Hz, 3H); ^{13}C NMR ($CDCl_3$, 100 MHz): δ 154.5, 143.4, 140.4, 140.1, 129.1, 127.9, 122.9, 115.5, 62.0, 42.3, 37.0, 21.7, 20.6, 14.0; HRMS (FAB) Calcd for $C_{16}H_{22}O_3S$ (M + H), 295.1368; found 295.1368.

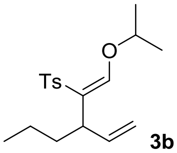

3b

^{1}H NMR ($CDCl_3$, 400 MHz): δ 7.81 (d, *J* = 8.5 Hz, 2H), 7.25 (d, *J* = 8.2 Hz, 2H), 6.31 (s, H), 5.79 (ddd, *J* = 17.1, 10.1, 7.4 Hz, H), 5.12 (d, *J* = 17.2 Hz, H), 5.07 (d, *J* = 10.5 Hz, H), 3.96 (septet, *J* = 6.2 Hz, H), 3.43 (q, *J* = 7.4 Hz, H), 2.42 (s, 3H), 1.69-1.52 (m, 2H), 1.42-1.33 (m, 2H), 1.11 (d, *J* = 6.2 Hz, 3H), 1.05 (d, *J* = 6.2 Hz, 3H), 0.93 (t, *J* =7.4 Hz, 3H); ^{13}C NMR ($CDCl_3$, 100 MHz): δ 151.9, 143.2, 140.8, 140.4, 128.8, 128.4, 122.7, 115.3, 78.3, 42.2, 37.1, 22.3, 22.2, 21.7, 20.7, 14.0; HRMS (FAB) Calcd for $C_{18}H_{26}O_3S$ (M + H), 323.1681; found 323.1682.

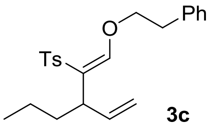

3c

^{1}H NMR ($CDCl_3$, 400 MHz): δ 7.78 (d, *J* = 8.2 Hz, 2H), 7.28-7.20 (m, 5H), 6.90 (d, *J* = 7.4 Hz, 2H), 6.08 (s, H), 5.61 (ddd, *J* = 17.6, 10.1, 7.8 Hz, H), 5.04 (d, *J* = 16.8 Hz, H), 5.01 (d, *J* = 9.8 Hz, H), 4.09 (dt, *J* = 10.6, 6.6 Hz, H), 4.04 (dt, *J* = 10.2, 7.0 Hz, H), 3.32 (q, *J* = 7.4 Hz, H), 2.79 (t, *J* = 7.0 Hz, 2H), 2.42 (s, 3H), 1.53-1.38 (m, 2H), 1.38-1.22 (m, 2H), 0.88 (t, *J* = 7.1 Hz, 3H); ^{13}C NMR ($CDCl_3$, 100 MHz): δ 153.6, 143.3, 140.5, 137.0, 129.1, 128.9, 128.7, 128.4, 128.3, 128.2, 126.8, 115.4, 76.2, 42.2, 36.9, 36.4, 21.7, 20.6, 14.0; HRMS (FAB) Calcd for $C_{23}H_{28}O_3S$ (M + H), 385.1837; found 385.1835.

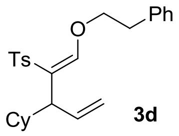

3d

^{1}H NMR ($CDCl_3$, 400 MHz): δ 7.77 (d, *J* = 8.2 Hz, 2H), 7.24-7.19 (m, 5H), 6.98 (d, *J* = 7.9 Hz, 2H), 6.08 (s, H), 5.68 (ddd, *J* = 17.1, 9.4, 8.5 Hz, H), 5.00 (d, *J* = 15.2 Hz, H), 5.00 (d, *J* = 11.3 Hz, H), 4.08 (dt, *J* = 10.1, 6.6 Hz, H), 4.02 (dt, *J* = 10.1, 7.0 Hz, H), 2.98 (t, *J* = 9.0 Hz, H), 2.77 (td, *J* = 6.6, 1.6 Hz, 2H), 2.41 (s, 3H), 1.77-1.62 (m, 4H), 1.52-1.44 (m, 4H), 1.25-1.05 (m, 4H), 0.89-0.75 (m, 2H); ^{13}C NMR ($CDCl_3$, 100 MHz): δ 153.5, 143.3, 140.2, 139.7, 137.0, 129.1, 128.9, 128.8, 128.2, 126.9, 121.4, 116.2, 76.3, 50.1, 40.9, 36.4, 31.5, 31.2, 26.5, 26.4, 26.3, 21.7; HRMS (FAB) Calcd for $C_{26}H_{32}O_3S$ (M + H), 425.2150; found 425.2151.

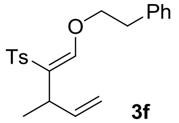

3f

^{1}H NMR ($CDCl_3$, 400 MHz): δ 7.79 (d, *J* = 8.2 Hz, 2H), 7.26-7.21 (m, 5H), 7.00 (d, *J* = 7.8 Hz, 2H), 6.08 (s, H), 5.68 (ddd, *J* = 16.4, 10.2, 5.9 Hz, H), 5.04 (d, *J* = 17.6 Hz, H), 5.00 (d, *J* = 10.5 Hz, H), 4.09 (dt, *J* = 10.1, 7.1 Hz, H), 4.06 (dt, *J* = 10.5, 6.7 Hz, H), 3.53 (quintet, *J* = 6.3 Hz, H), 2.80 (t, *J* = 6.6 Hz, 2H), 2.42 (s, 3H), 1.18 (d, *J* = 7.0 Hz, 3H); ^{13}C NMR ($CDCl_3$, 100 MHz): δ 153.7, 143.5, 141.0, 140.0, 137.0, 129.1, 129.0, 128.7, 128.1, 126.8, 122.6, 114.5, 76.3, 36.4, 36.0, 21.7, 20.2; HRMS (FAB) Calcd for $C_{21}H_{24}O_3S$ (M + H), 357.1524; found 357.1526.

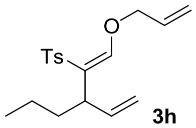

3h

^{1}H NMR ($CDCl_3$, 400 MHz): δ 7.82 (d, *J* = 8.2 Hz, 2H), 7.26 (d, *J* = 8.6 Hz, 2H), 6.27 (s, H), 5.75 (ddd, *J* = 17.6, 10.2, 7.4 Hz, H), 5.67 (ddt, *J* = 17.2, 11.7, 5.1 Hz, H), 5.17 (dd, *J* = 15.6, 1.1 Hz, H), 5.14 (dd, *J* = 16.0, 1.2 Hz, H), 5.09 (d, *J* = 11.0 Hz, H), 5.06 (d, *J* = 11.1 Hz, H), 4.34-4.33 (m, 2H), 3.40 (q, *J* = 7.5 Hz, H), 2.43 (s, 3H), 1.66-1.50 (m, 2H), 1.41-1.26 (m, 2H), 0.91 (t, *J* = 7.5 Hz, 3H); ^{13}C NMR ($CDCl_3$, 100 MHz): δ 152.8, 143.4, 140.5, 140.0, 132.2, 129.0, 128.2, 123.1, 118.9, 115.5, 75.2, 42.3, 37.0, 21.7, 20.6, 14.0; HRMS (FAB) Calcd for $C_{18}H_{24}O_3S$ (M + H), 321.1524; found 321.1525.

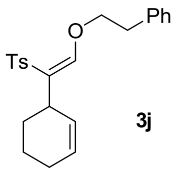

3j

Inseparable mixture with **4**, **5** (R = $(CH_2)_2Ph$) and $Ph(CH_2)_2OH$

^{1}H NMR ($CDCl_3$, 400 MHz): δ 7.78 (d, *J* = 8.2 Hz, 2H), 7.35-7.18 (m, 5H), 7.00 (d, *J* = 7.9 Hz, 2H), 6.10 (s, H), 5.77-5.72 (m, H), 5.27-5.21 (m, H), 4.02 (t, *J* = 7.2 Hz, 2H), 3.55-3.46 (m, H), 2.79 (t, *J* = 7.0 Hz, 2H), 2.43 (s, 3H), 2.01-1.87 (m, 4H), 1.58-1.38 (m, 2H). LRMS (EI^+, GC-MS) Calcd for $C_{23}H_{27}O_3S$ (M + H), 383; found 383.

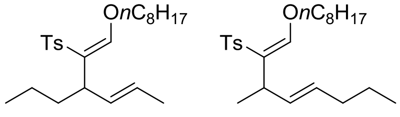

3k **3k'**

3k and **3k'** (1:1.4), inseparable mixture with **4** (R = C_8H_{17})

^{1}H NMR ($CDCl_3$, 400 MHz): δ 6.28 (s, H, **3k'**), 6.26 (s, H, **3k**), 5.58-5.29 (m, 2H, **3k** and **3k'**), 3.64 (t, *J* = 6.6 Hz, 2H, **3k** and **3k'**), 3.53-3.40 (m, H, **3k**), 3.33 (app q, *J* = 7.1 Hz, H, **3k'**). LRMS (EI^+, GC-MS) Calcd for $C_{24}H_{39}O_3S$ (M + H), 407; found 407.

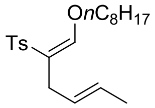

3l

^{1}H NMR ($CDCl_3$, 400 MHz): δ 7.81 (d, *J* = 8.2 Hz, 2H), 7.26 (d, *J* = 8.2 Hz, 2H), 6.29 (s, H), 5.60-5.48 (m, H), 5.45-5.35 (m, H), 3.84 (t, *J* = 6.7 Hz, 2H), 3.04 (d, *J* = 5.8 Hz, 2H), 2.42 (s, 3H), 1.67 (d, *J* = 5.5 Hz, 3H), 1.56-1.48 (m, 2H), 1.36-1.12 (m, 10H), 0.89 (t, *J* = 6.7 Hz, 3H); ^{13}C NMR ($CDCl_3$, 100 MHz): δ 153.0, 143.1, 139.7, 128.7, 127.8, 127.7, 127.4, 117.6, 75.3, 31.6, 31.2, 29.4, 29.0, 28.9, 25.3, 22.4, 21.4, 17.6, 13.9; HRMS (FAB) Calcd for $C_{21}H_{33}O_3S$ (M + H), 365.2150; found 365.2151.

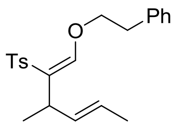

3m

^{1}H NMR ($CDCl_3$, 400 MHz): δ 7.77 (d, *J* = 7.8 Hz, 2H), 6.99 (d, *J* = 7.1 Hz, 2H), 6.05 (s, H), 5.50-5.39 (m, H), 5.28-5.19 (m, H), 4.02 (t, *J* = 7.0 Hz, 2H), 3.52-3.45 (m, H), 3.00 (t, *J* = 7.0 Hz, 2H), 2.42 (s, 3H), 1.62 (d, *J* = 6.2 Hz, 3H), 1.15 (d, *J* = 7.0 Hz, 3H). LRMS (EI^+, GC-MS) Calcd for $C_{22}H_{27}O_3S$ (M + H), 371; found 371.

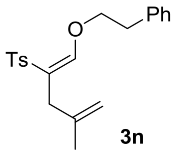

3n

^{1}H NMR ($CDCl_3$, 400 MHz): δ 7.77 (d, *J* = 8.2 Hz, 2H), 7.26-7.22 (m, 5H), 7.06 (d, *J* = 6.3 Hz, 2H), 6.21 (s, 1H), 4.82 (s, 1H), 4.76 (s, 1H), 4.10 (t, *J* = 6.6 Hz, 2H), 2.98 (s, 2H), 2.85 (t, *J* = 7.0 Hz, 2H), 2.42 (s, 3H), 1.60 (s, 3H); ^{13}C NMR ($CDCl_3$, 100 MHz): δ 153.7, 143.4, 142.3, 139.6, 136.9, 129.0, 128.6, 128.0, 126.8, 116.6, 113.4, 76.1, 36.2, 21.7, 21.6; HRMS (FAB) Calcd for $C_{21}H_{24}O_3S$ (M + H), 357.1524; found 357.1537.

4. Scanned copies of the spectra for substrates

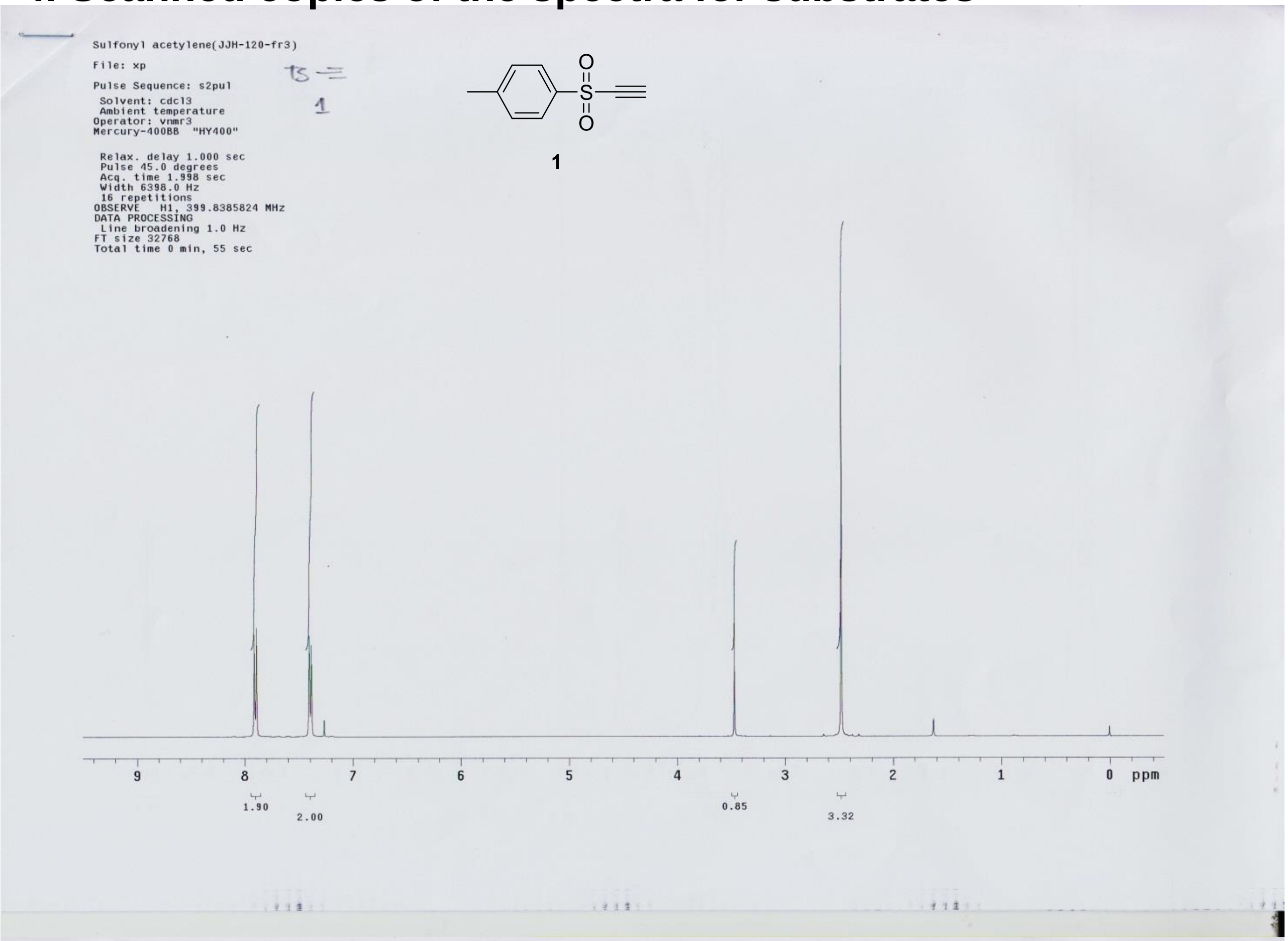

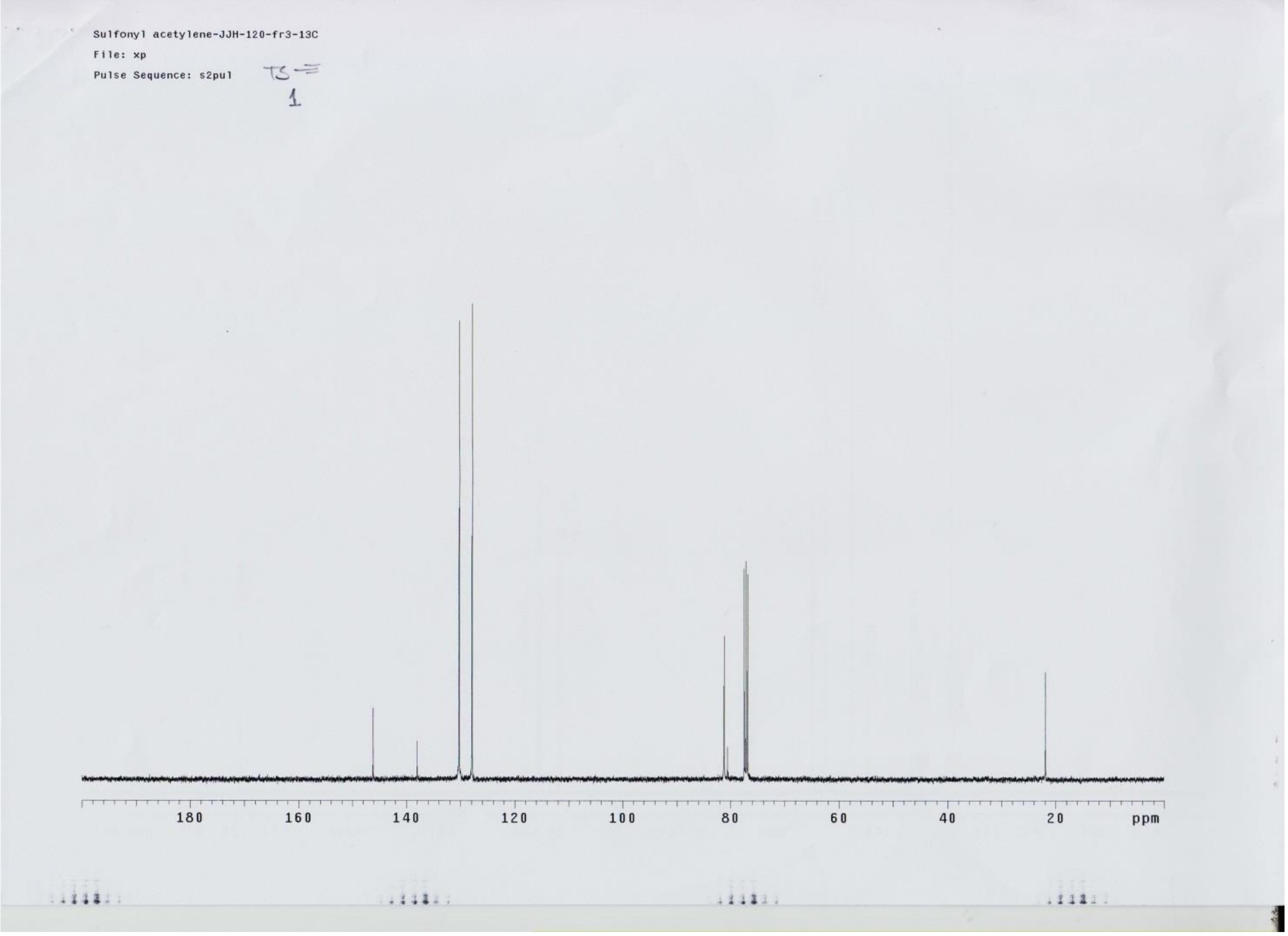

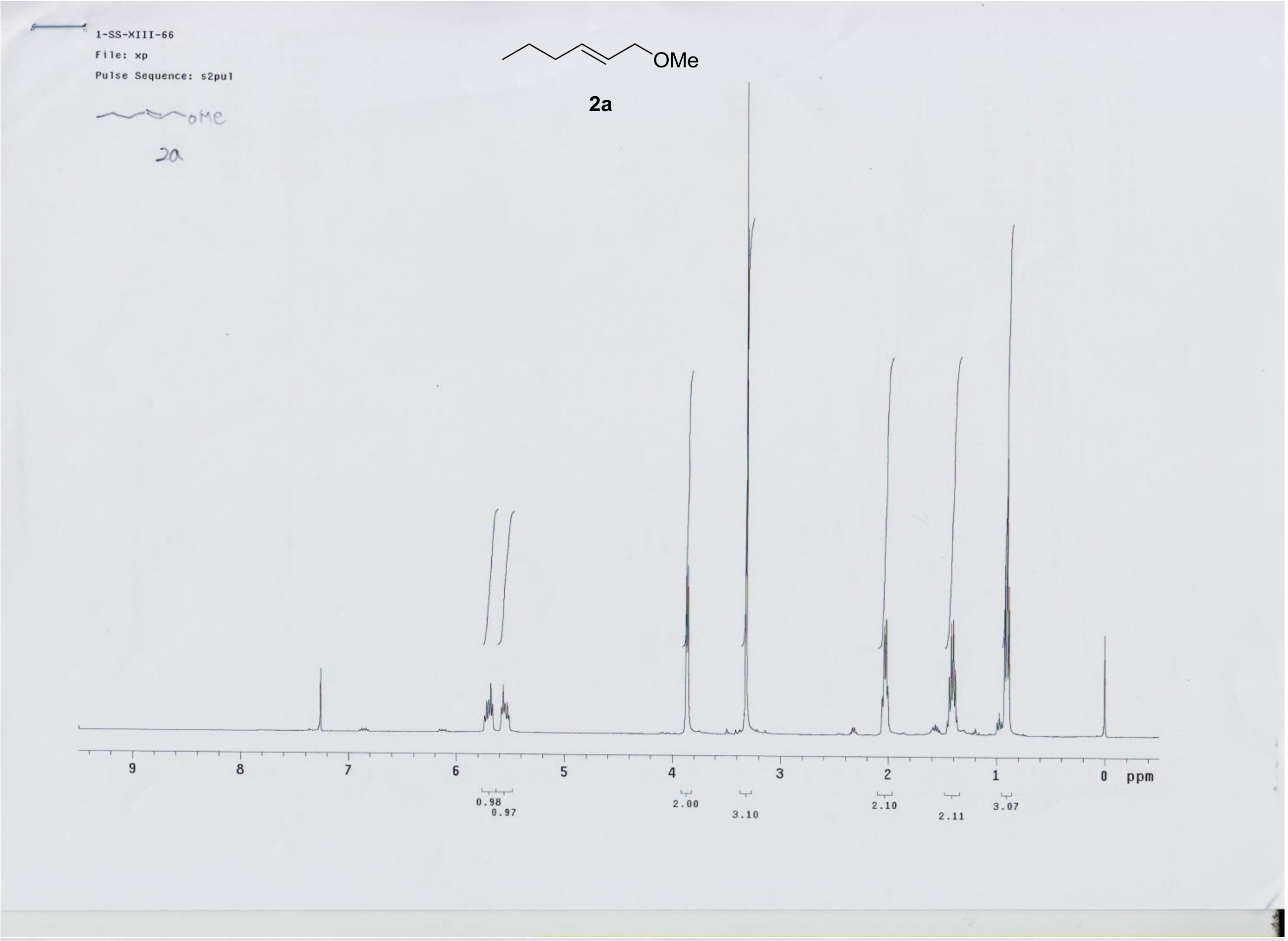
1-SS-XIII-66
File: xp
Pulse Sequence: s2pul
OMe
2a
9 8 7 6 5 4 3 2 1 0 ppm
0.98
0.97
2.00
3.10
2.10
2.11
3.07

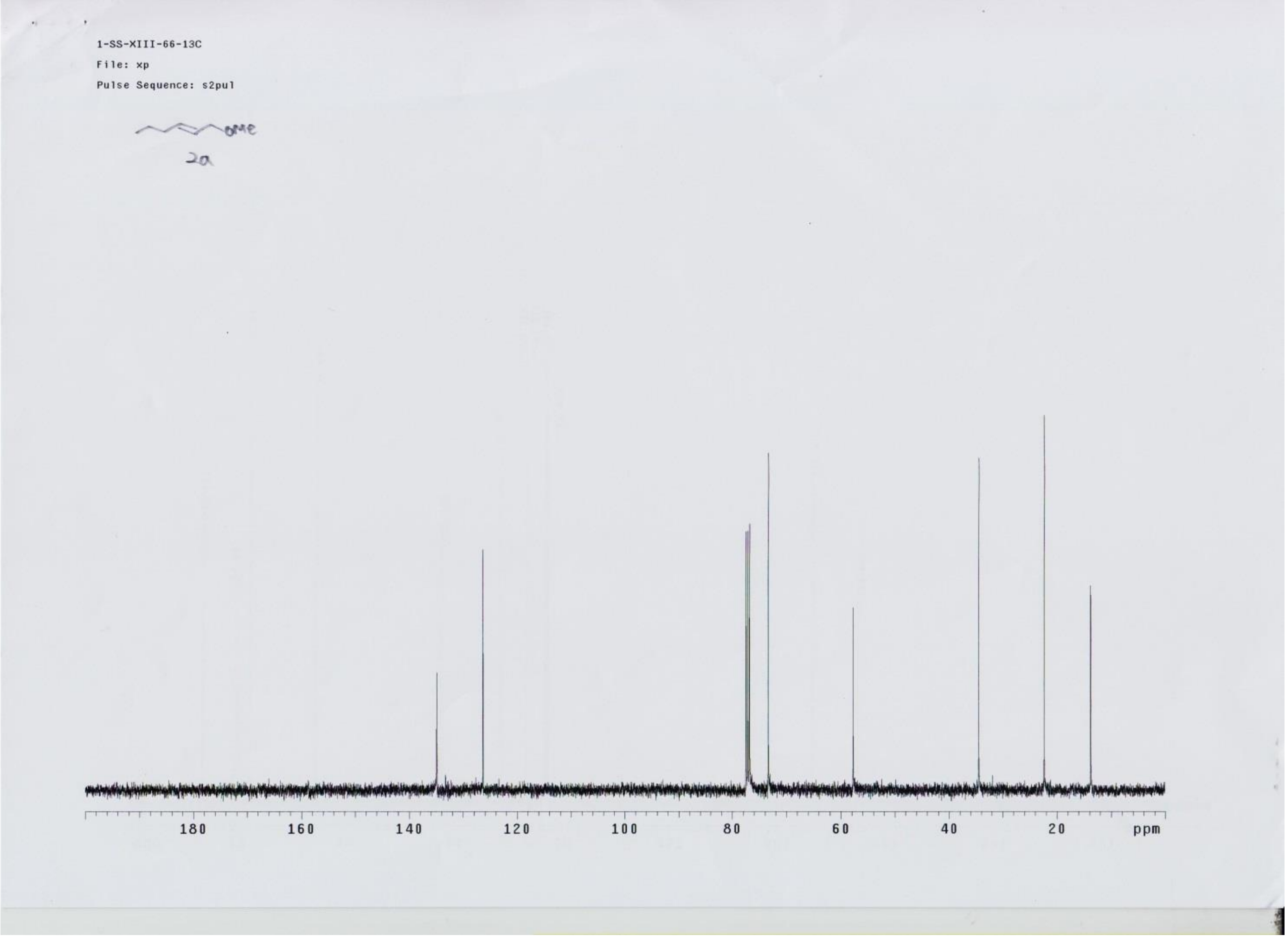
1-SS-XIII-66-13C
File: xp
Pulse Sequence: s2pul
180 160 140 120 100 80 60 40 20 ppm

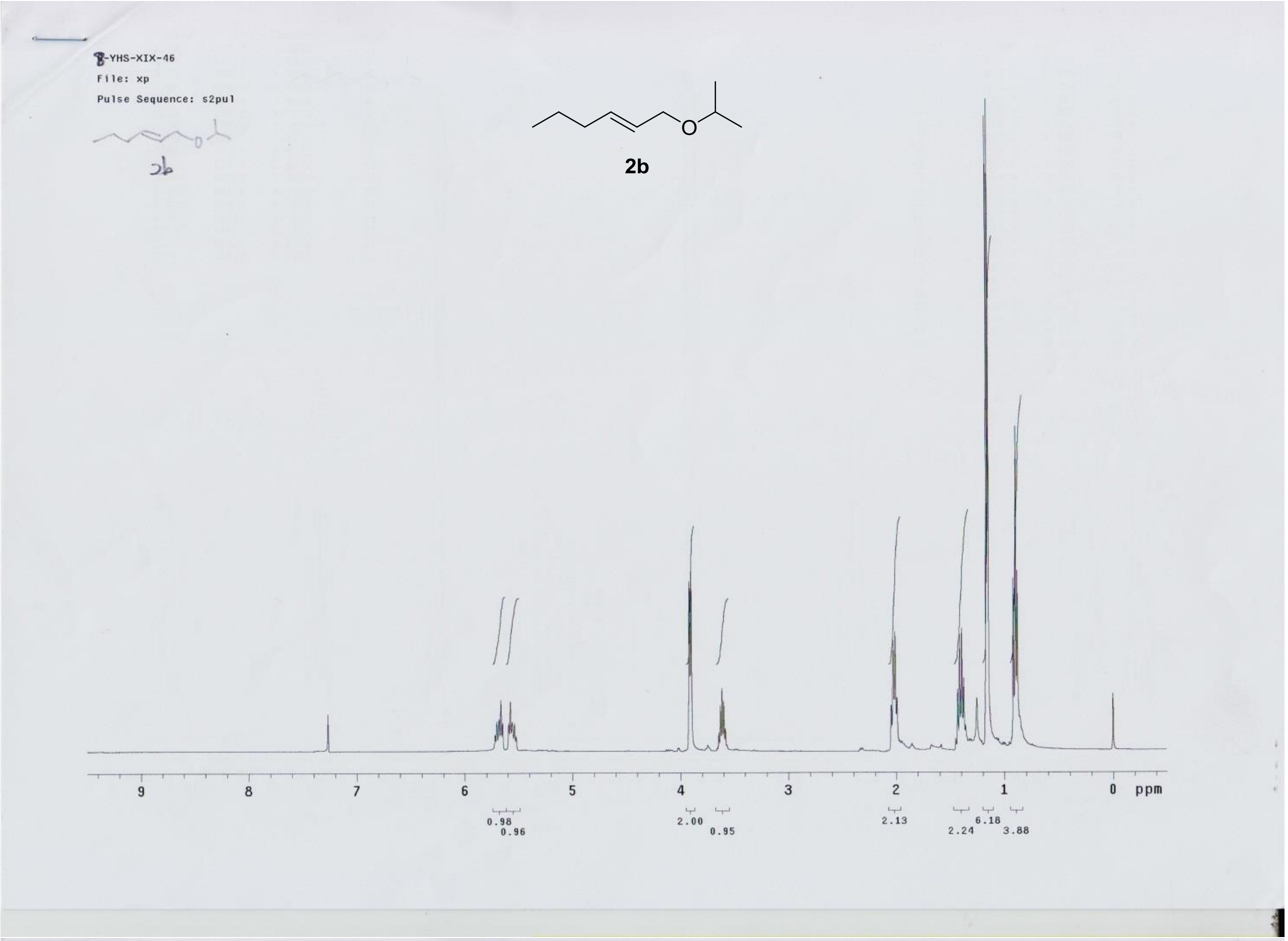
8-YHS-XIX-46
File: xp
Pulse Sequence: s2pul
2b
9 8 7 6 5 4 3 2 1 0 ppm
0.98
0.96
2.00
0.95
2.13
2.24
6.18
3.88

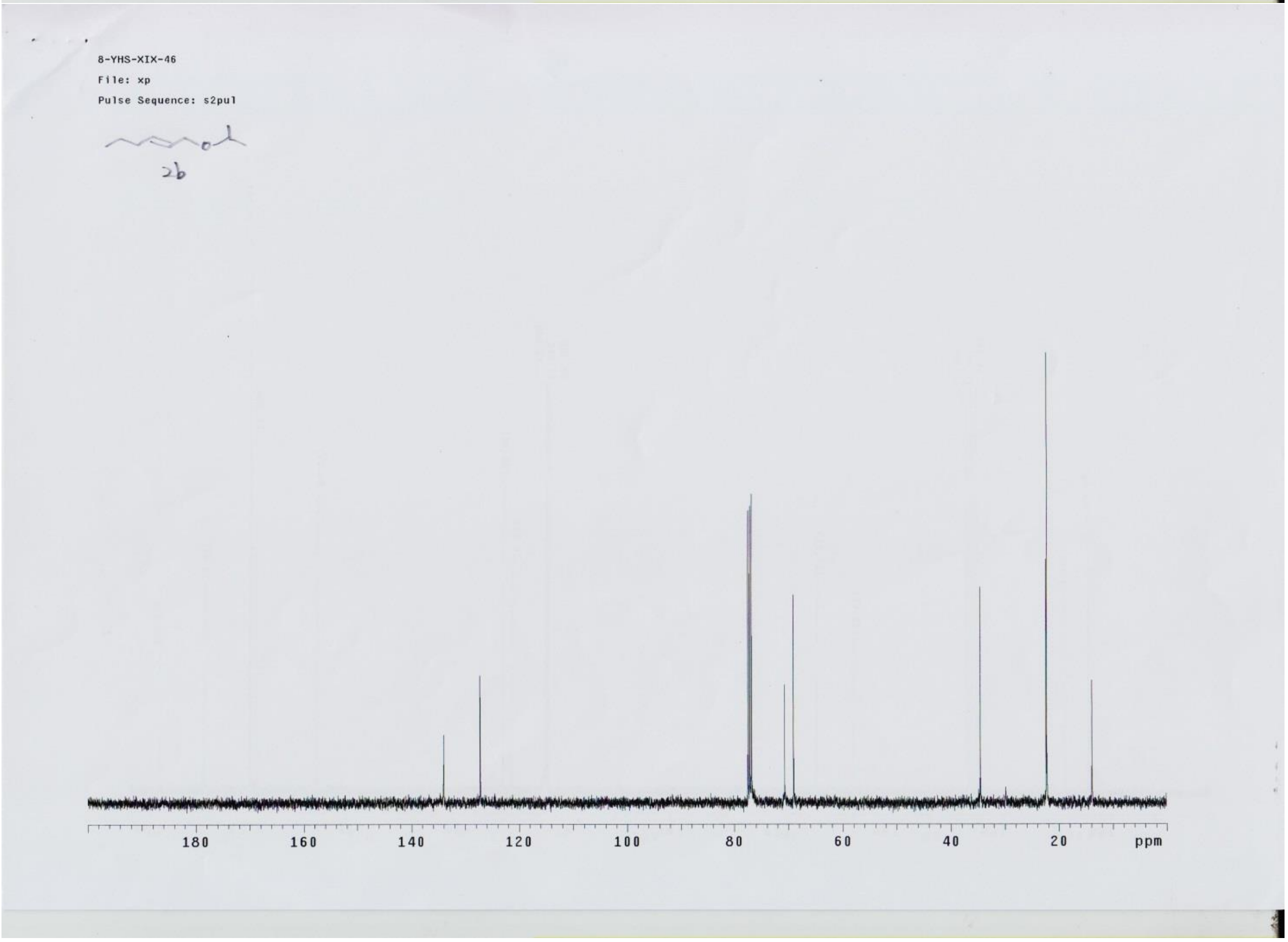
8-YHS-XIX-46
File: xp
Pulse Sequence: s2pul
2b
180 160 140 120 100 80 60 40 20 ppm

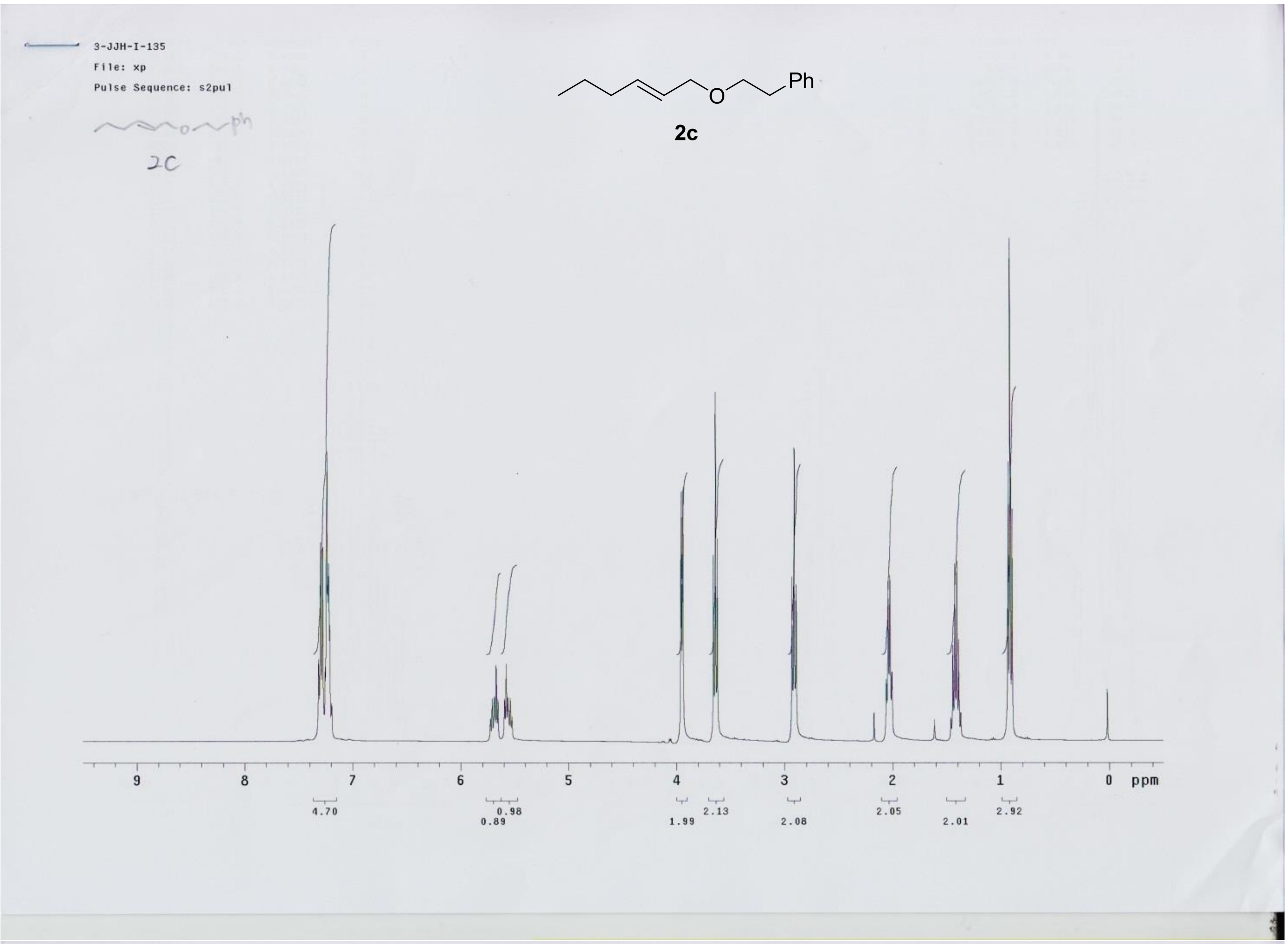
3-JJH-I-135
File: xp
Pulse Sequence: s2pul
Ph
O
2c
9
8
7
6
5
4
3
2
1
0
ppm
4.70
0.98
0.89
1.99
2.13
2.08
2.05
2.01
2.92

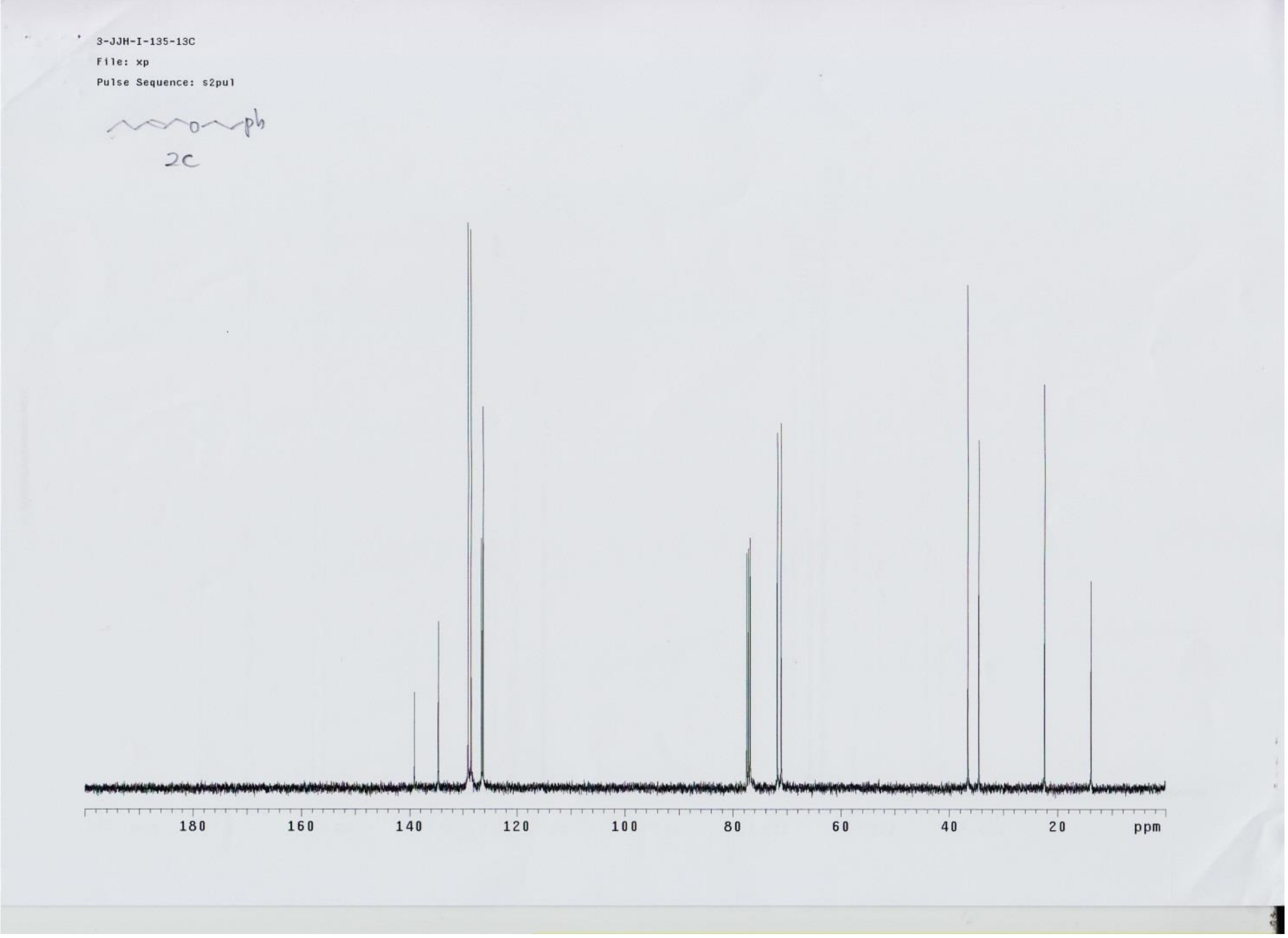
3-JJH-I-135-13C
File: xp
Pulse Sequence: s2pul
180
160
140
120
100
80
60
40
20
ppm

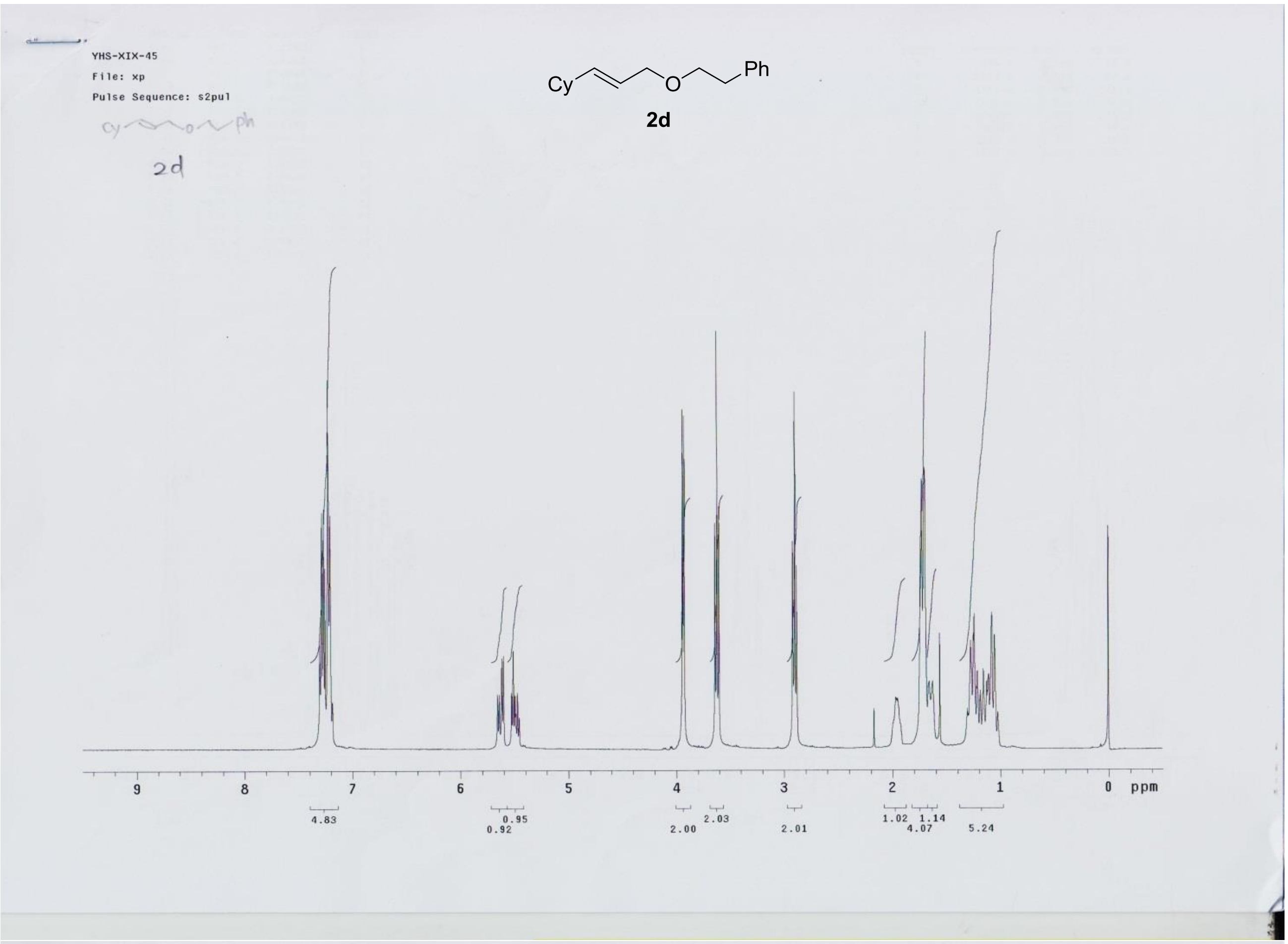
YHS-XIX-45
File: xp
Pulse Sequence: s2pul
Cy O Ph
2d
9 8 7 6 5 4 3 2 1 0 ppm
4.83
0.92 0.95
2.00 2.03
2.01
1.02 1.14 4.07
5.24

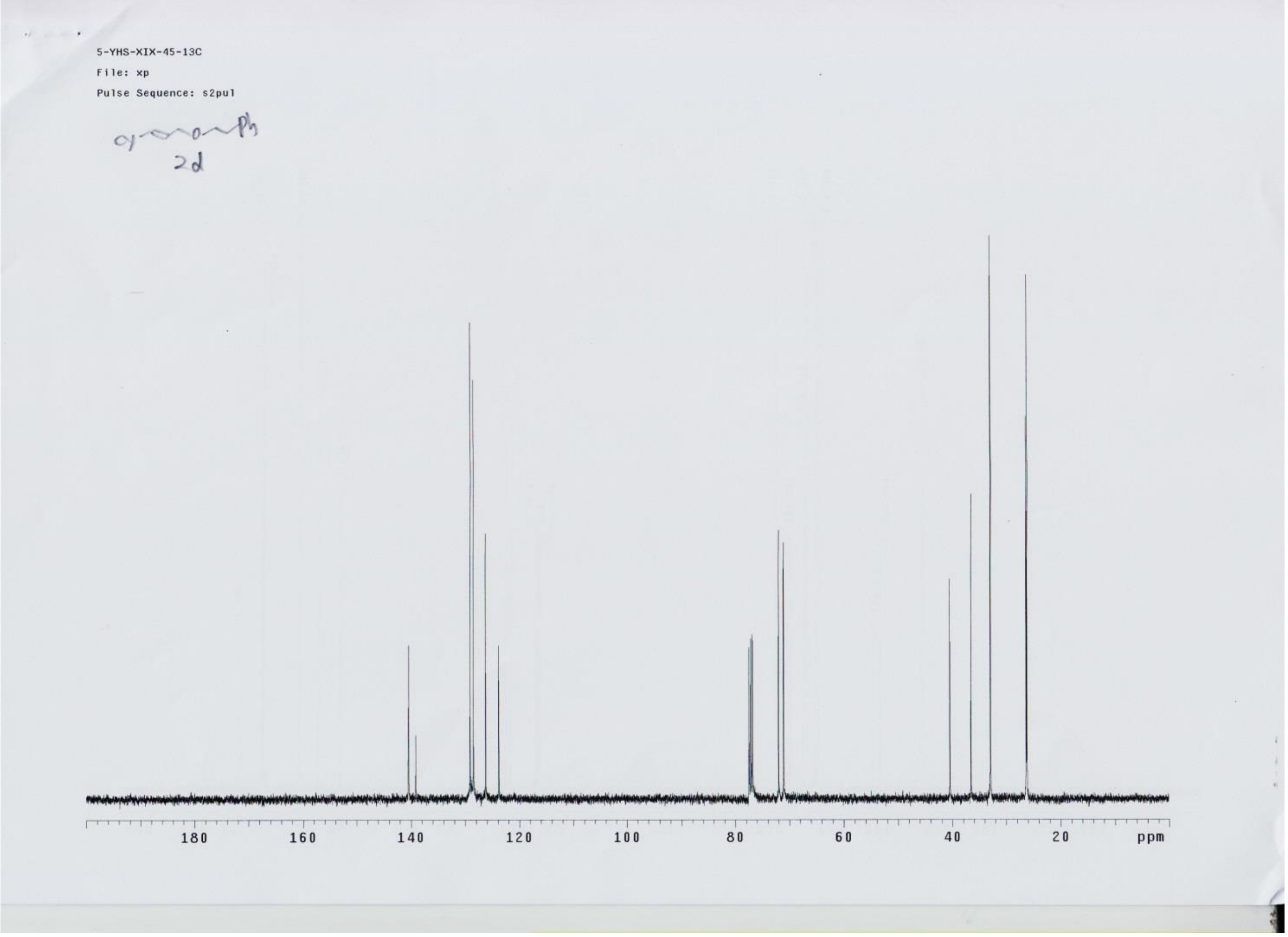
5-YHS-XIX-45-13C
File: xp
Pulse Sequence: s2pul
2d
180 160 140 120 100 80 60 40 20 ppm

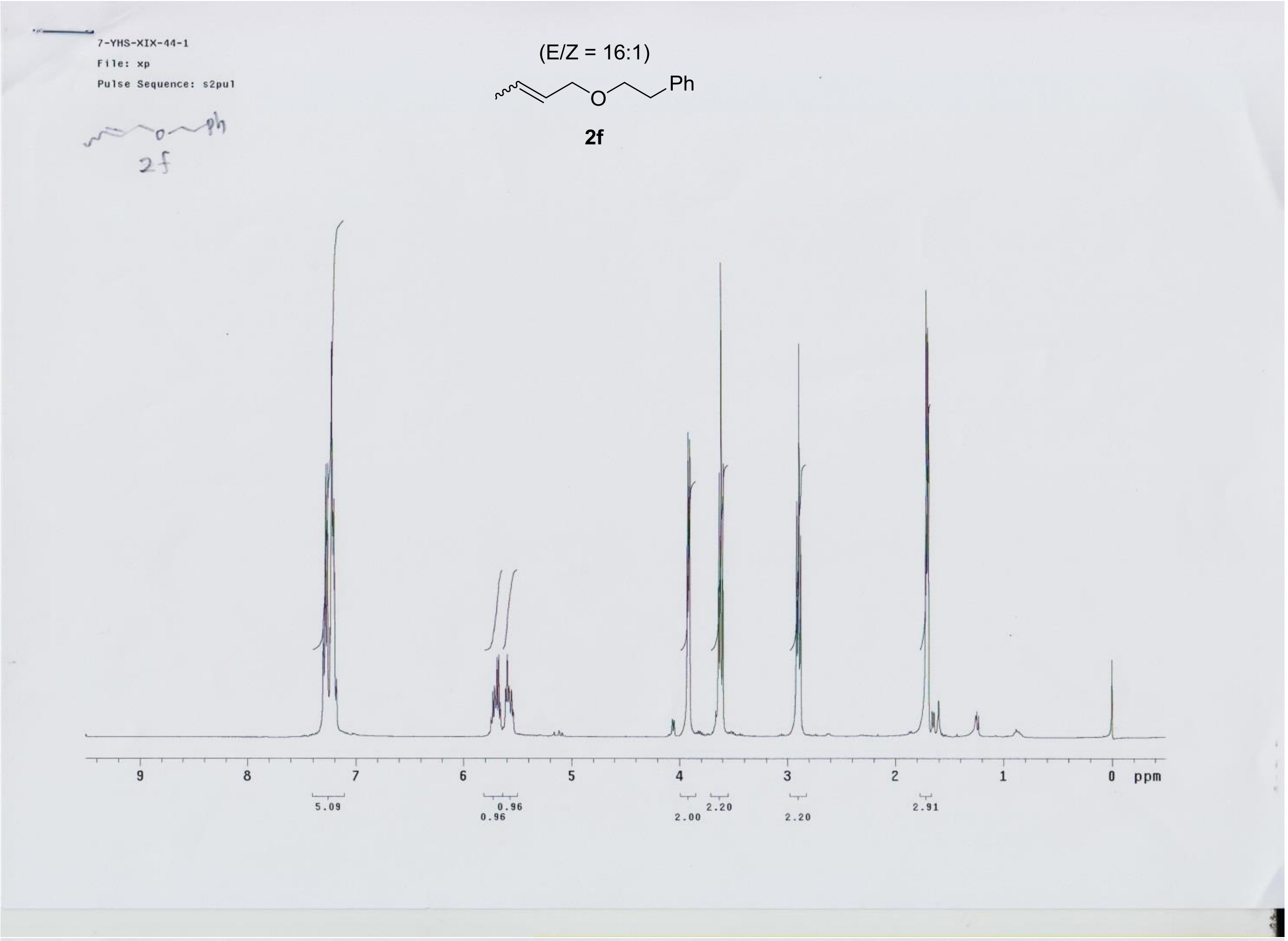
7-YHS-XIX-44-1
File: xp
Pulse Sequence: s2pul
(E/Z = 16:1)
Ph
O
2f
9 8 7 6 5 4 3 2 1 0 ppm
5.09
0.96
0.96
2.00
2.20
2.20
2.91

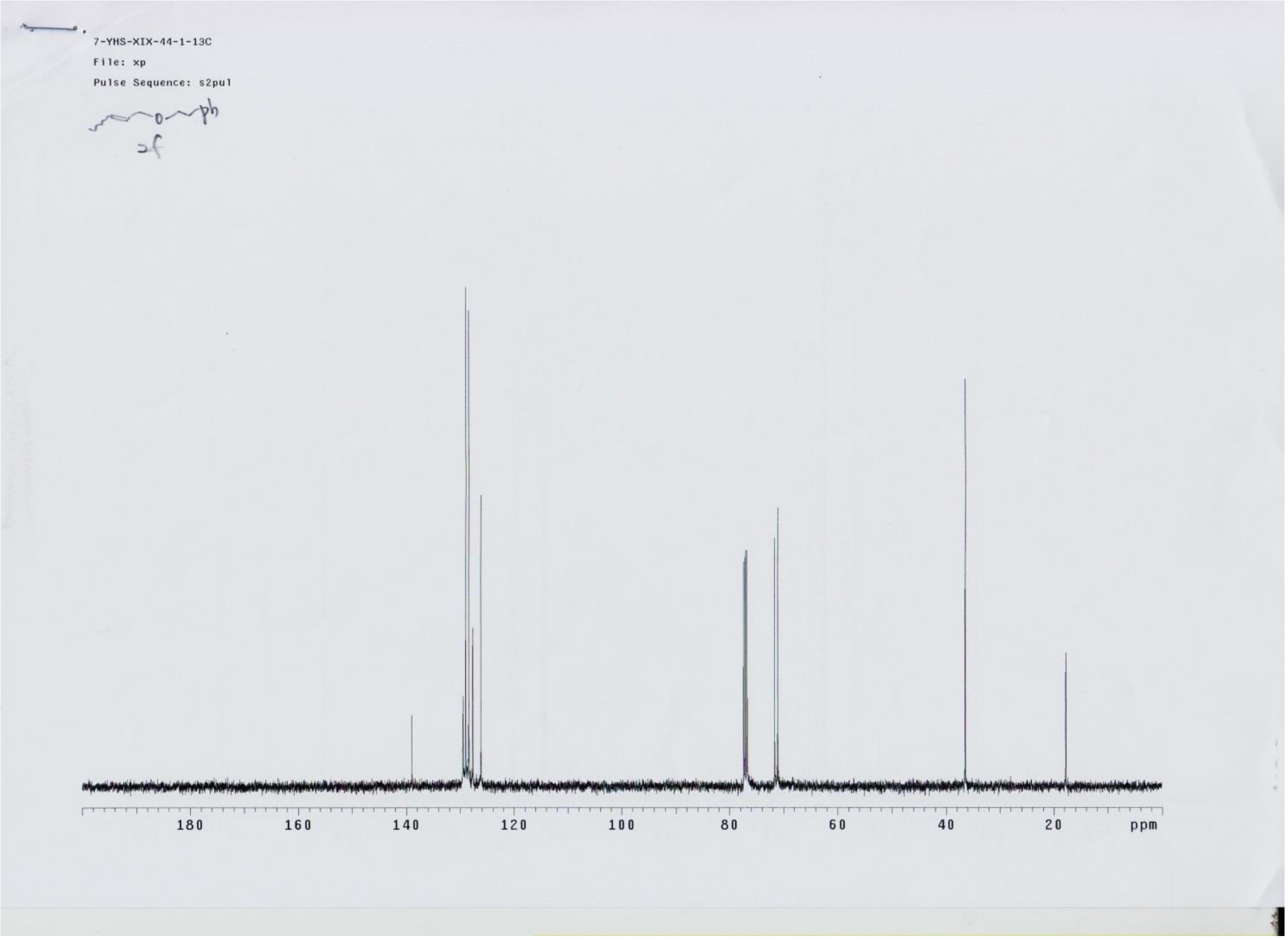
7-YHS-XIX-44-1-13C
File: xp
Pulse Sequence: s2pul
180 160 140 120 100 80 60 40 20 ppm

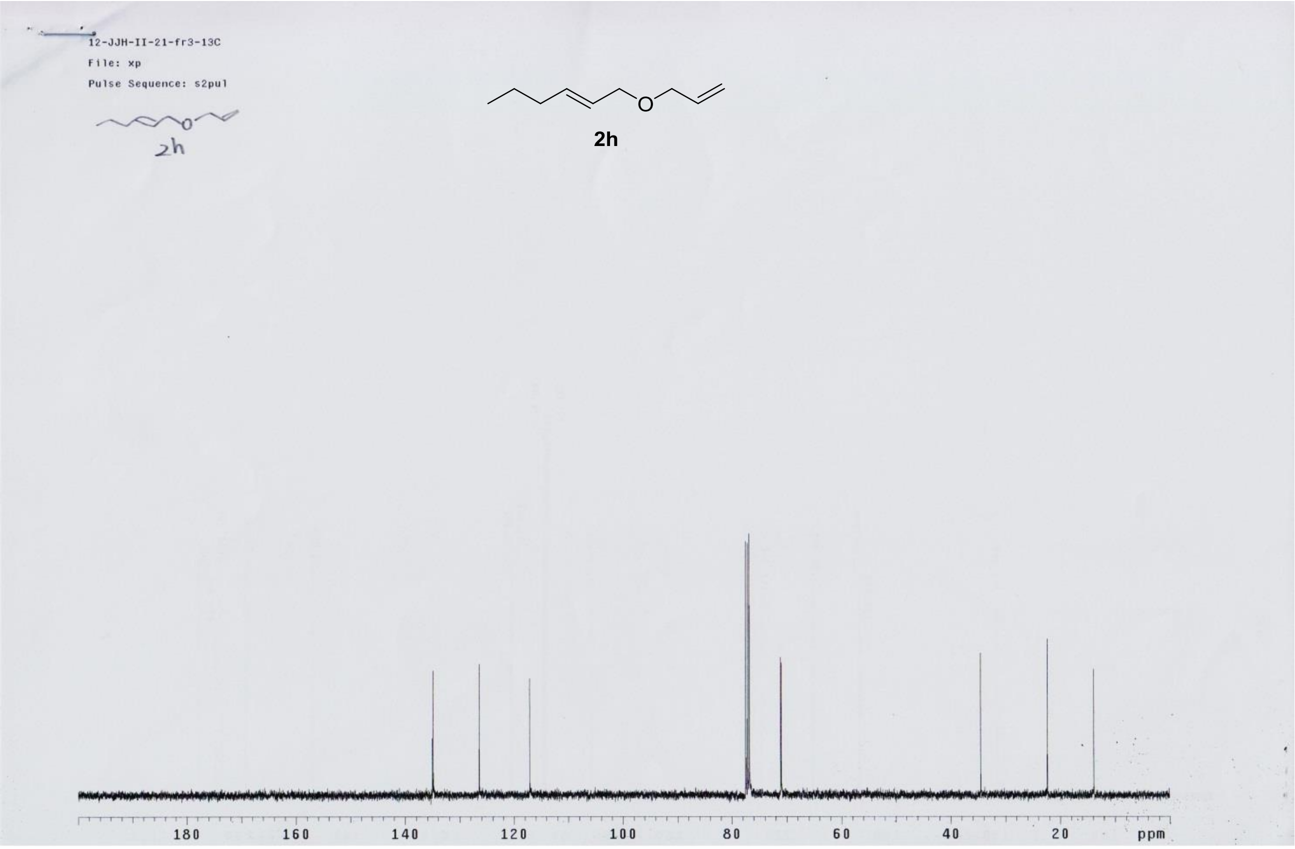
12-JJH-II-21-fr3-13C
File: xp
Pulse Sequence: s2pul
2h
180 160 140 120 100 80 60 40 20 ppm

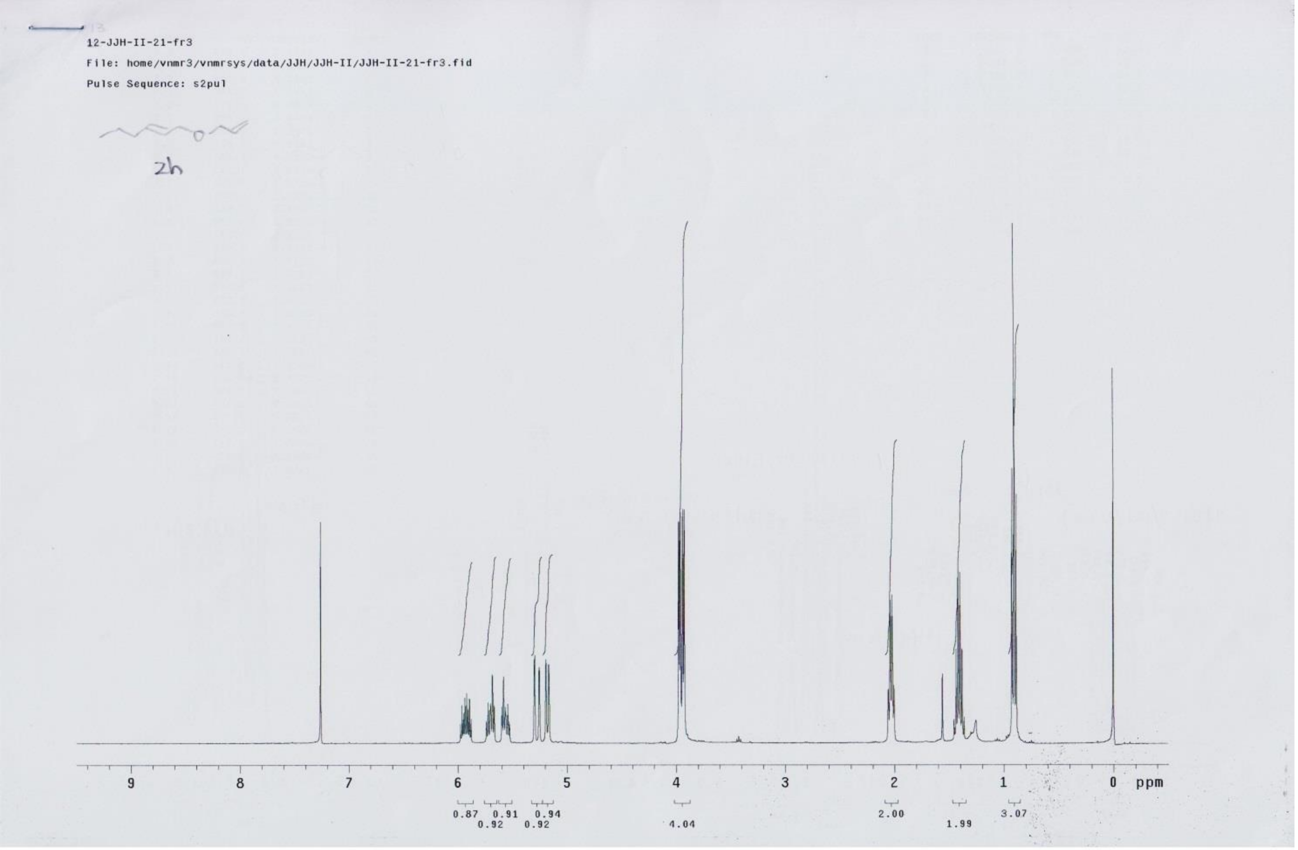
12-JJH-II-21-fr3
File: home/vnmr3/vnmrsys/data/JJH/JJH-II/JJH-II-21-fr3.fid
Pulse Sequence: s2pul
2h
9 8 7 6 5 4 3 2 1 0 ppm
0.87 0.92 0.91 0.92 0.94 4.04 2.00 1.99 3.07

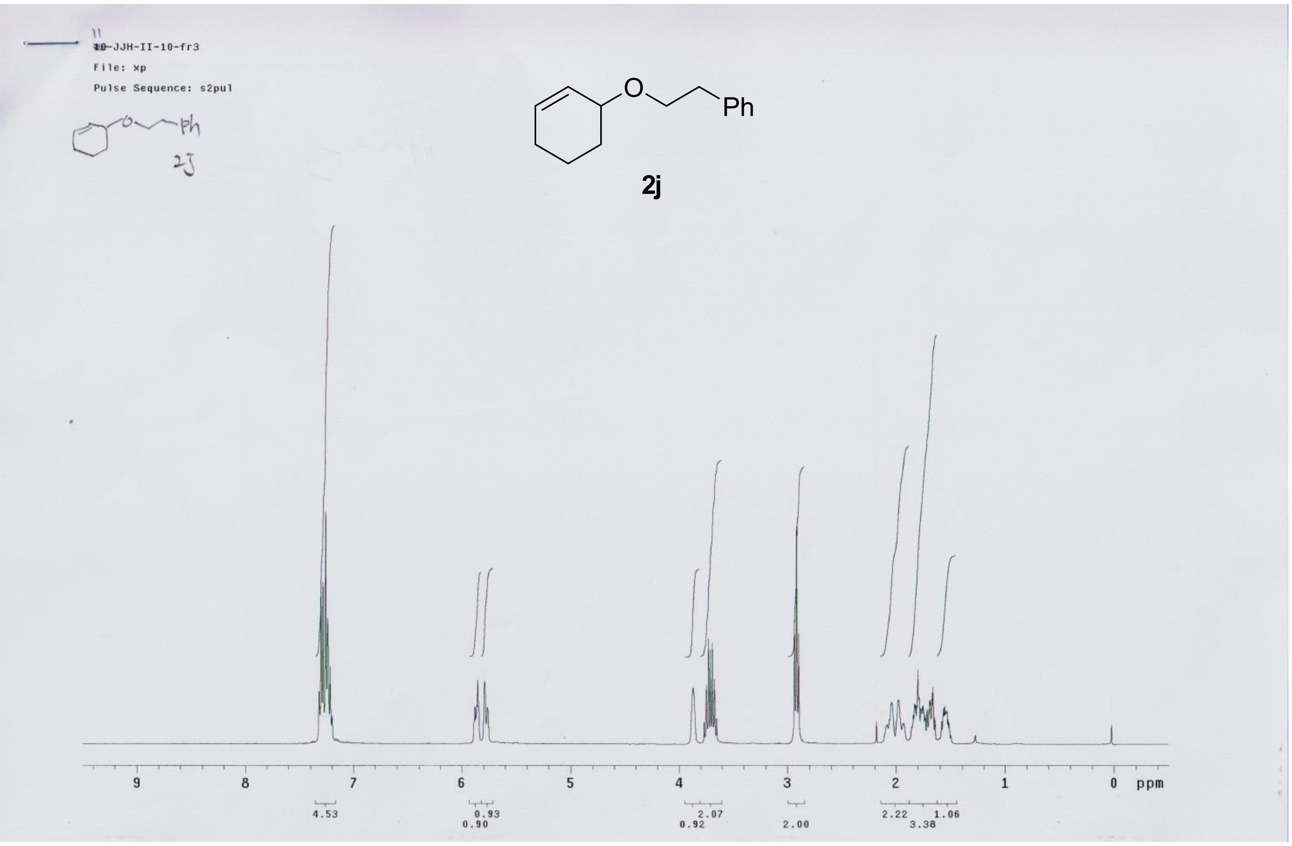
11-JJH-II-10-fr3
File: xp
Pulse Sequence: s2pul
Ph
2j
9
8
7
6
5
4
3
2
1
0
ppm
4.53
0.93
0.90
2.07
0.92
2.00
2.22
1.06
3.38

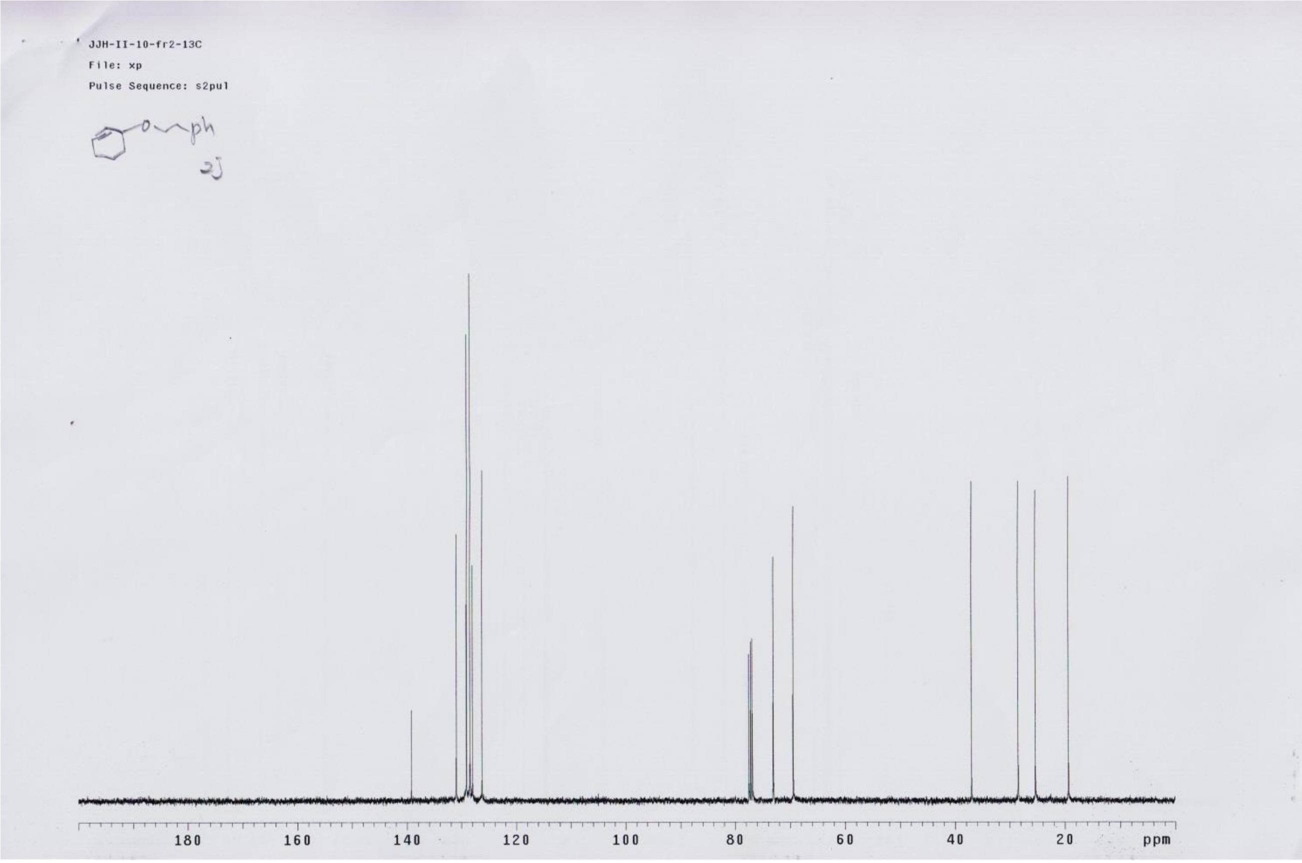
JJH-II-10-fr2-13C
File: xp
Pulse Sequence: s2pul
ph
2j
180
160
140
120
100
80
60
40
20
ppm

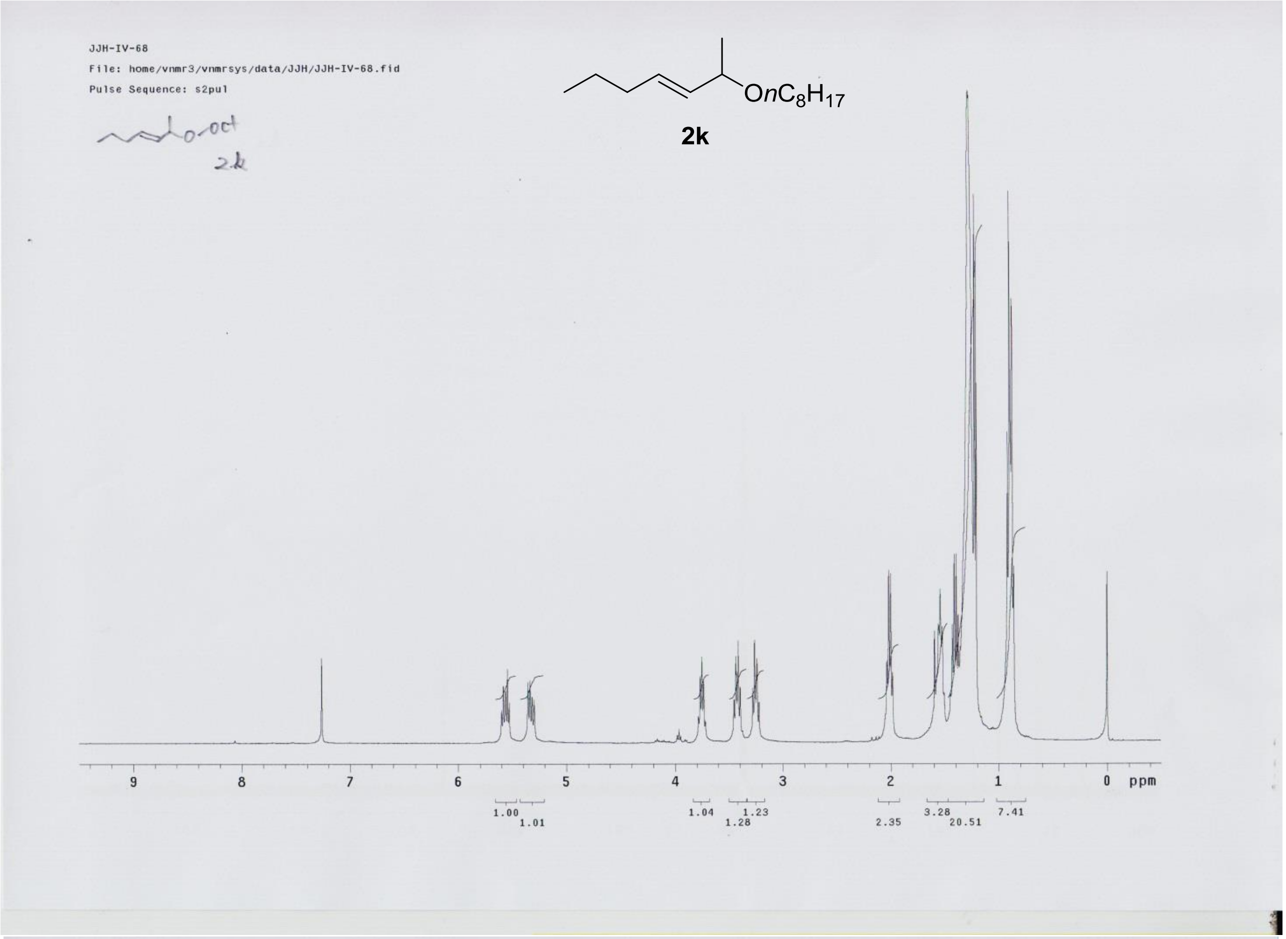
JJH-IV-68
File: home/vnmr3/vnmrsys/data/JJH/JJH-IV-68.fid
Pulse Sequence: s2pul
OnC_8H_{17}
2k
ppm

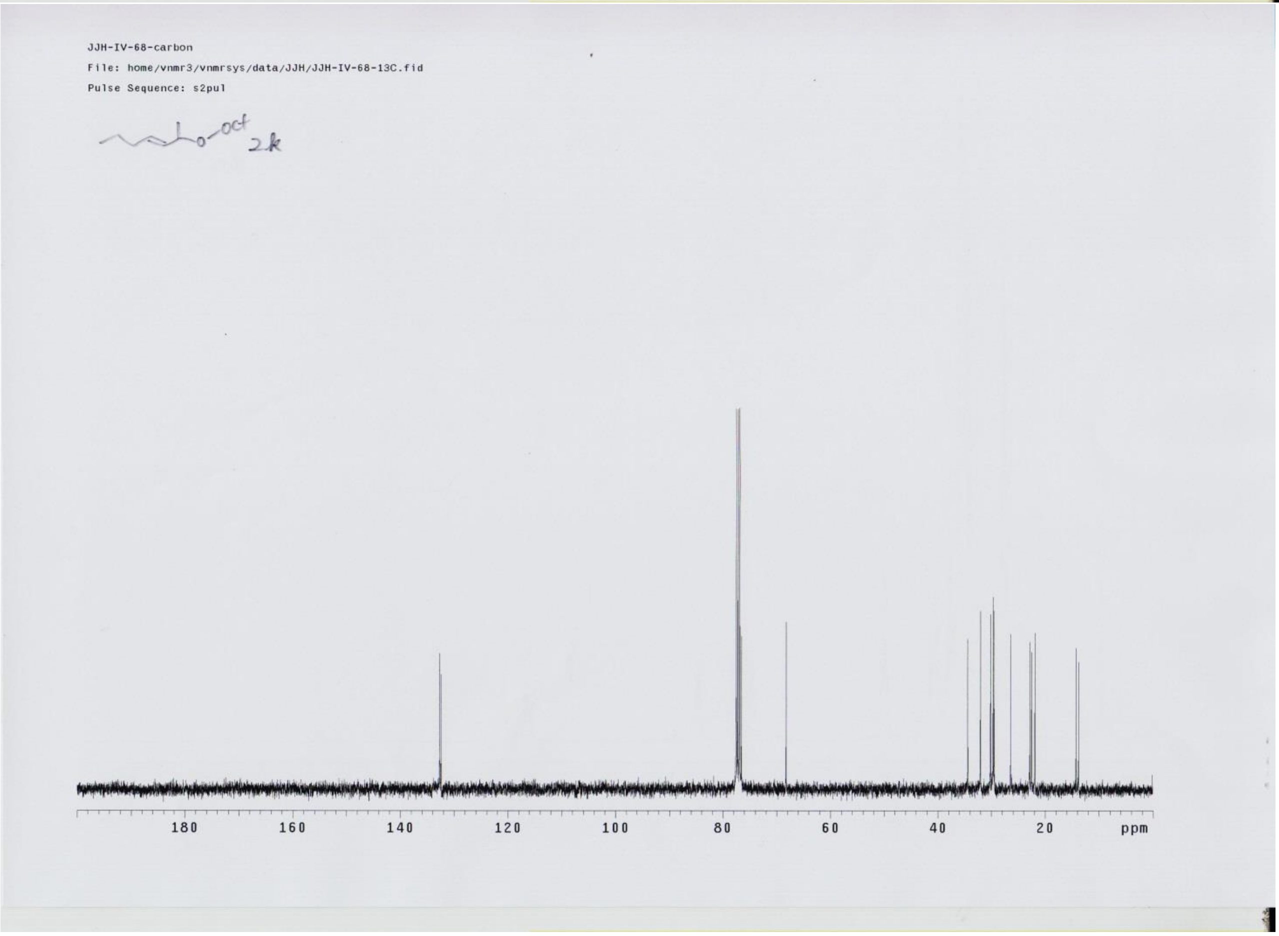
JJH-IV-68-carbon
File: home/vnmr3/vnmrsys/data/JJH/JJH-IV-68-13C.fid
Pulse Sequence: s2pul
ppm

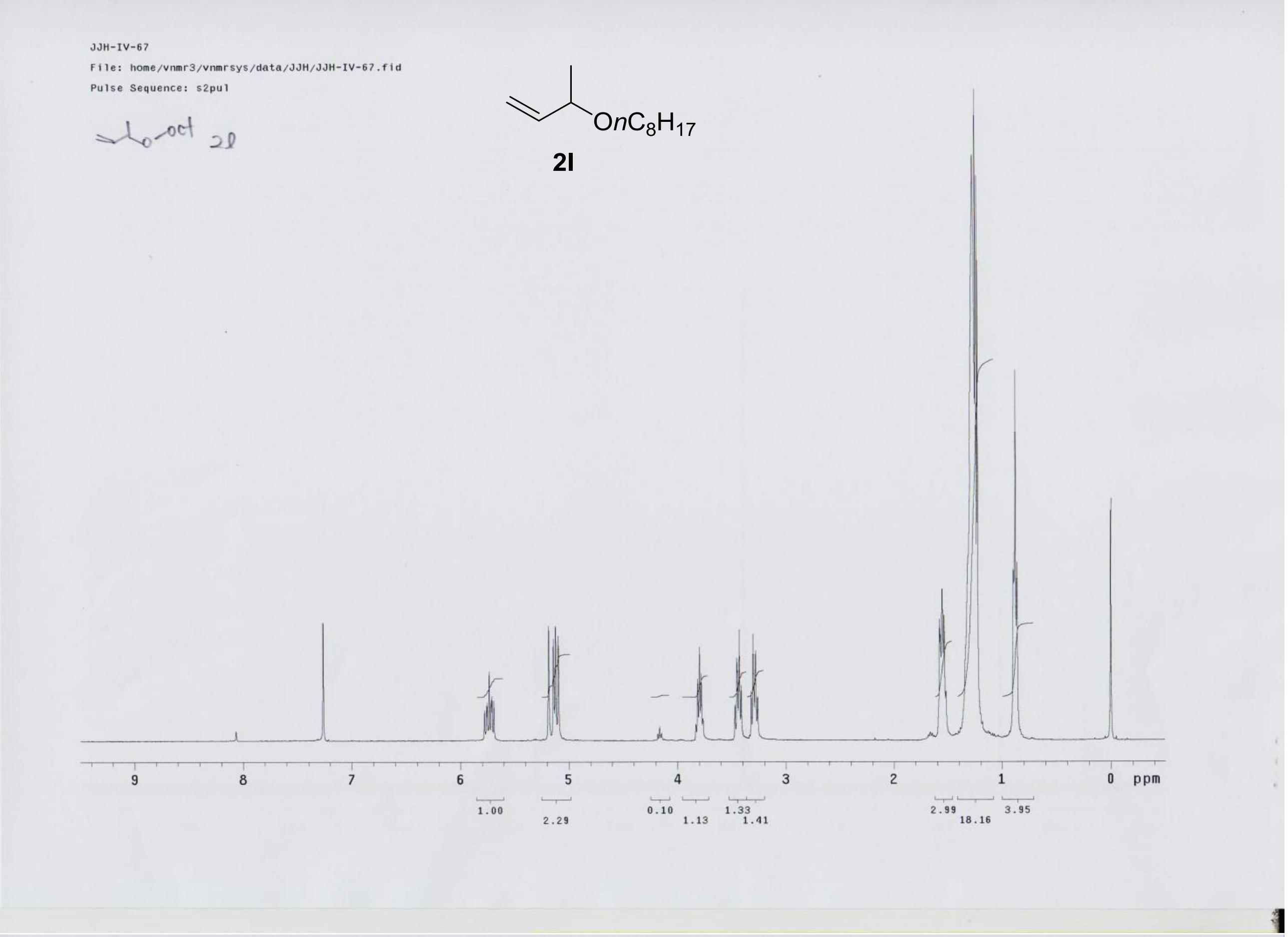
JJH-IV-67
File: home/vnmr3/vnmrsys/data/JJH/JJH-IV-67.fid
Pulse Sequence: s2pul
OnC8H17
2l
9 8 7 6 5 4 3 2 1 0 ppm
1.00
2.29
0.10
1.13
1.33
1.41
2.99
18.16
3.95

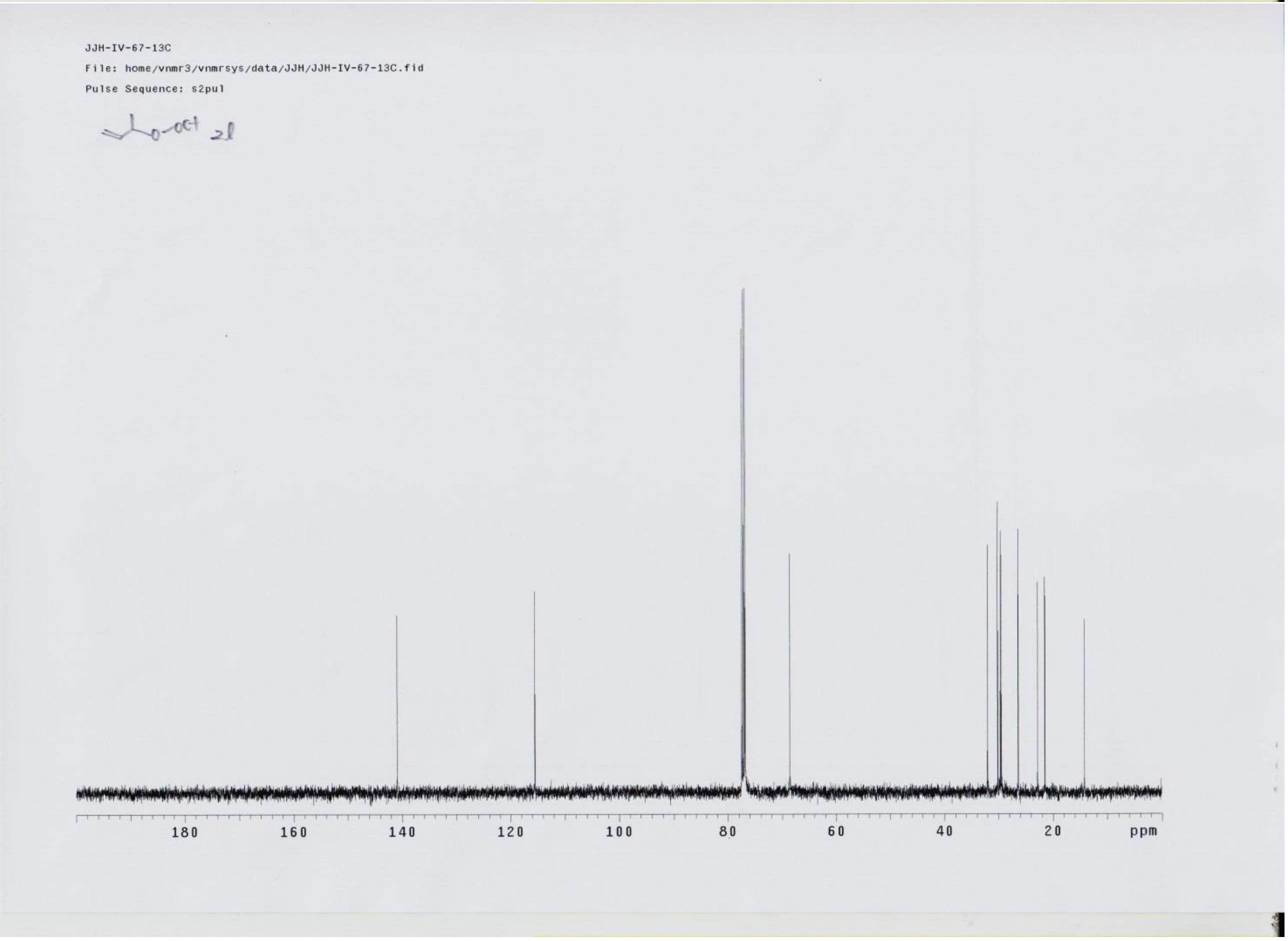
JJH-IV-67-13C
File: home/vnmr3/vnmrsys/data/JJH/JJH-IV-67-13C.fid
Pulse Sequence: s2pul
180 160 140 120 100 80 60 40 20 ppm

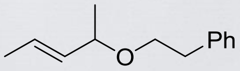

2m

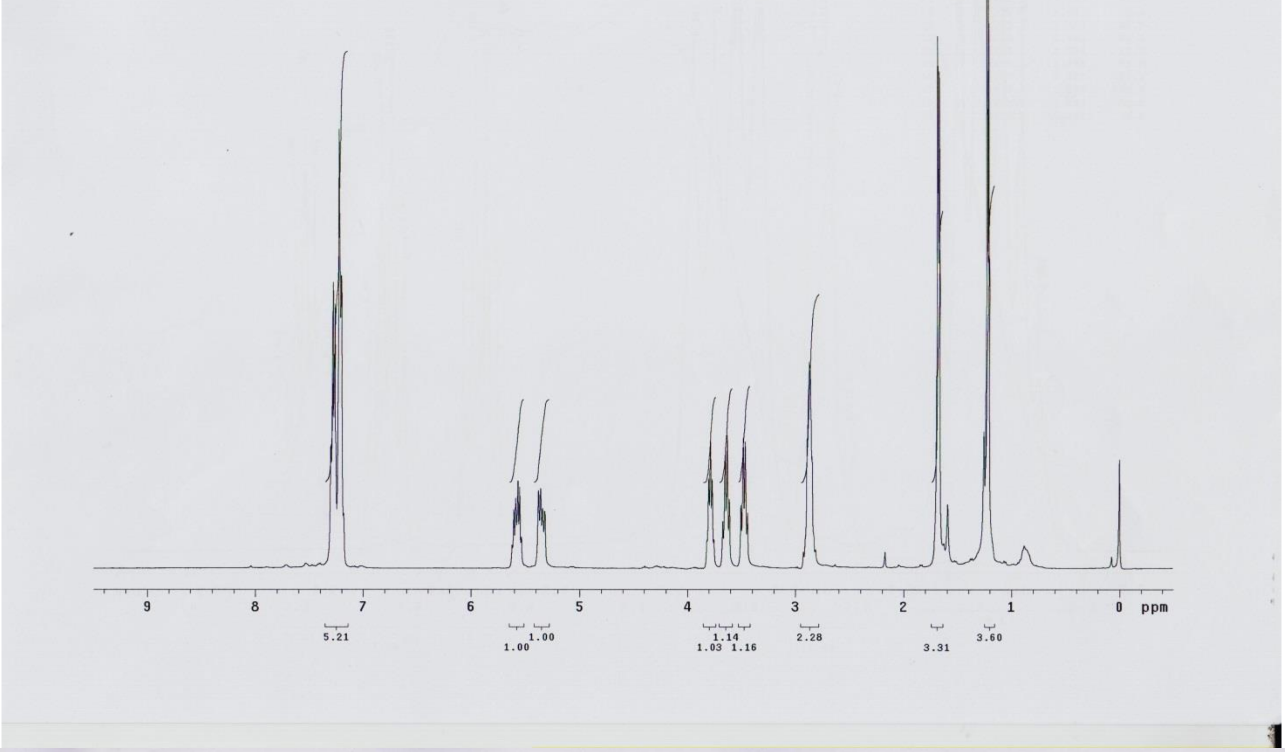
JJH-II-112-fr1
File: xp
Pulse Sequence: s2pul
9 8 7 6 5 4 3 2 1 0 ppm
5.21
1.00
1.00
1.14
1.03 1.16
2.28
3.31
3.60

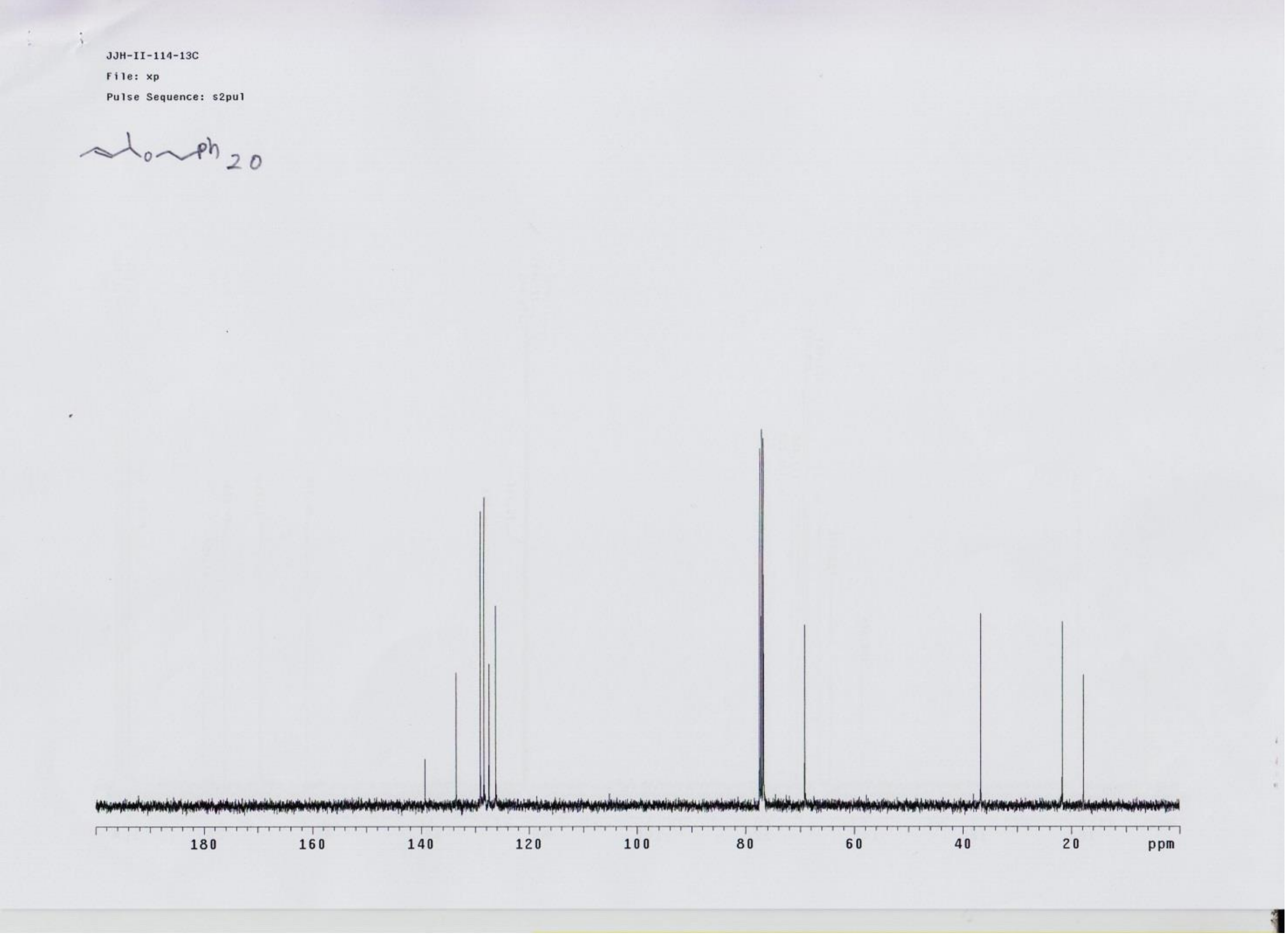
JJH-II-114-13C
File: xp
Pulse Sequence: s2pul
180 160 140 120 100 80 60 40 20 ppm

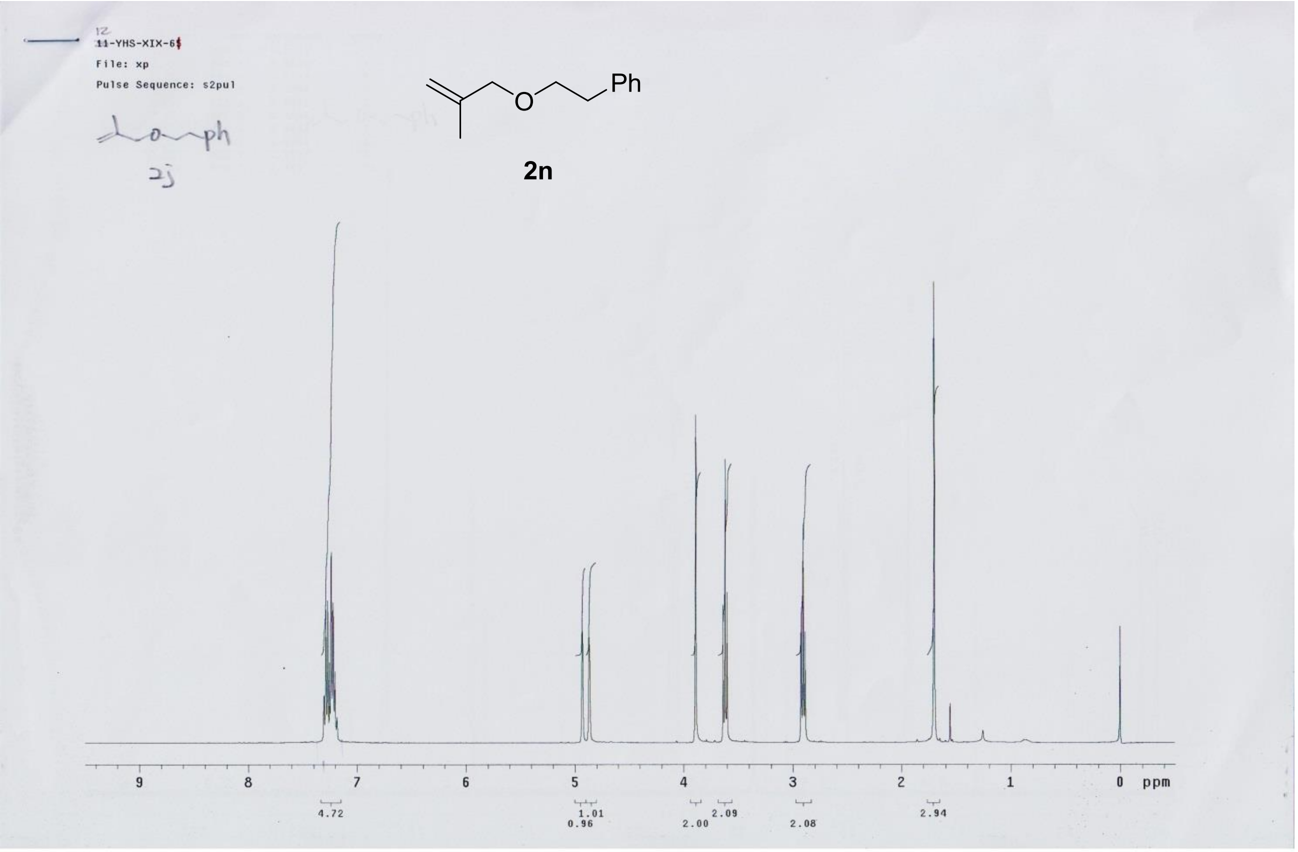
File: xp
Pulse Sequence: s2pul
Ph
2n

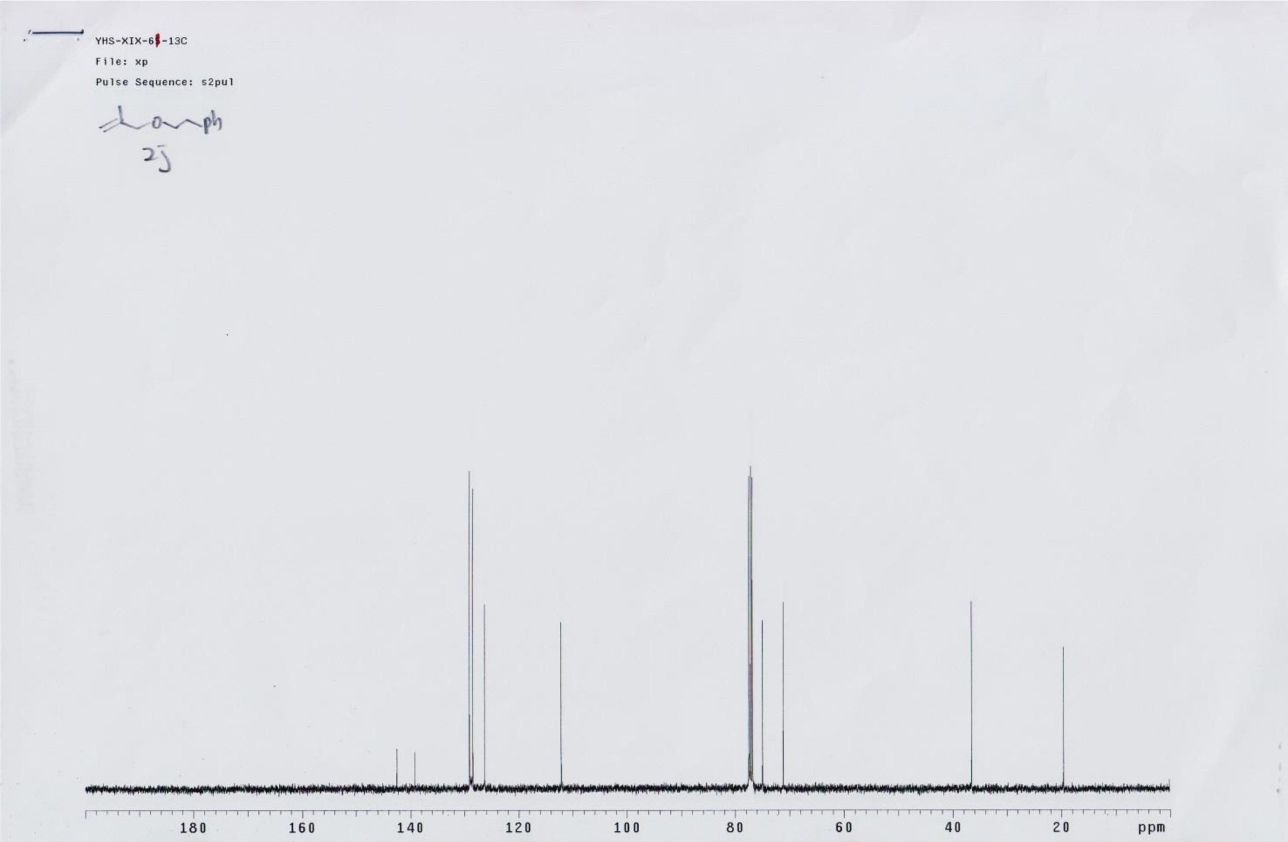
File: xp
Pulse Sequence: s2pul

5. Scanned copies of the spectra for products

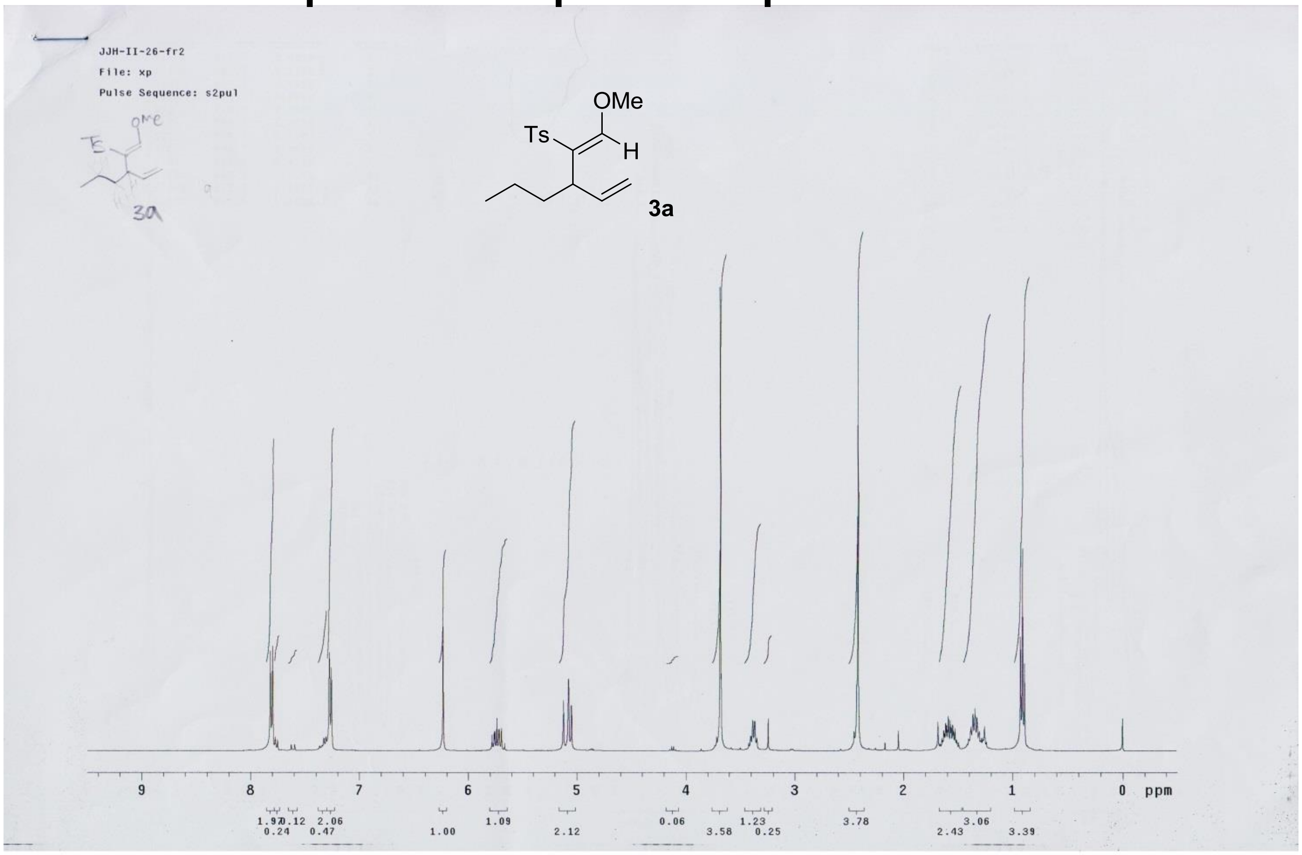

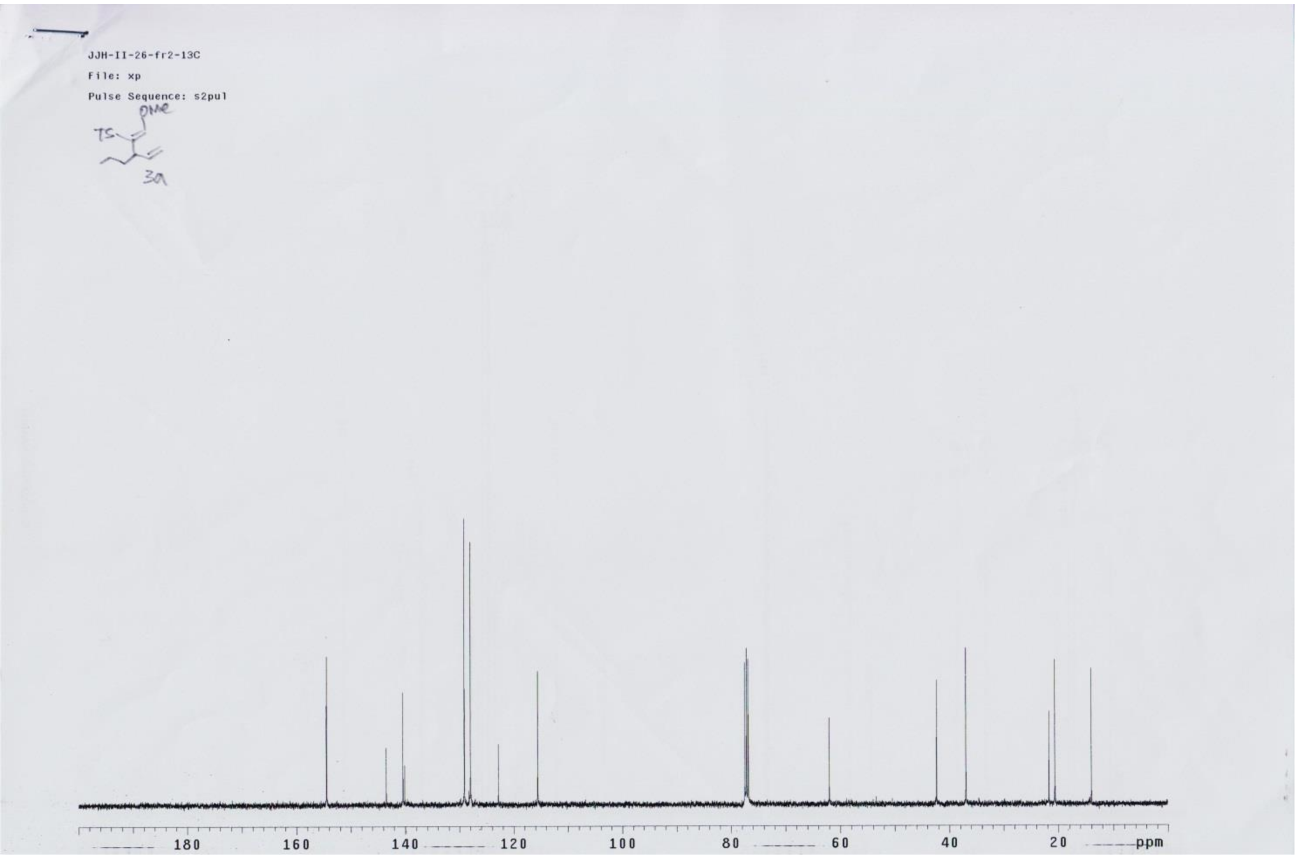

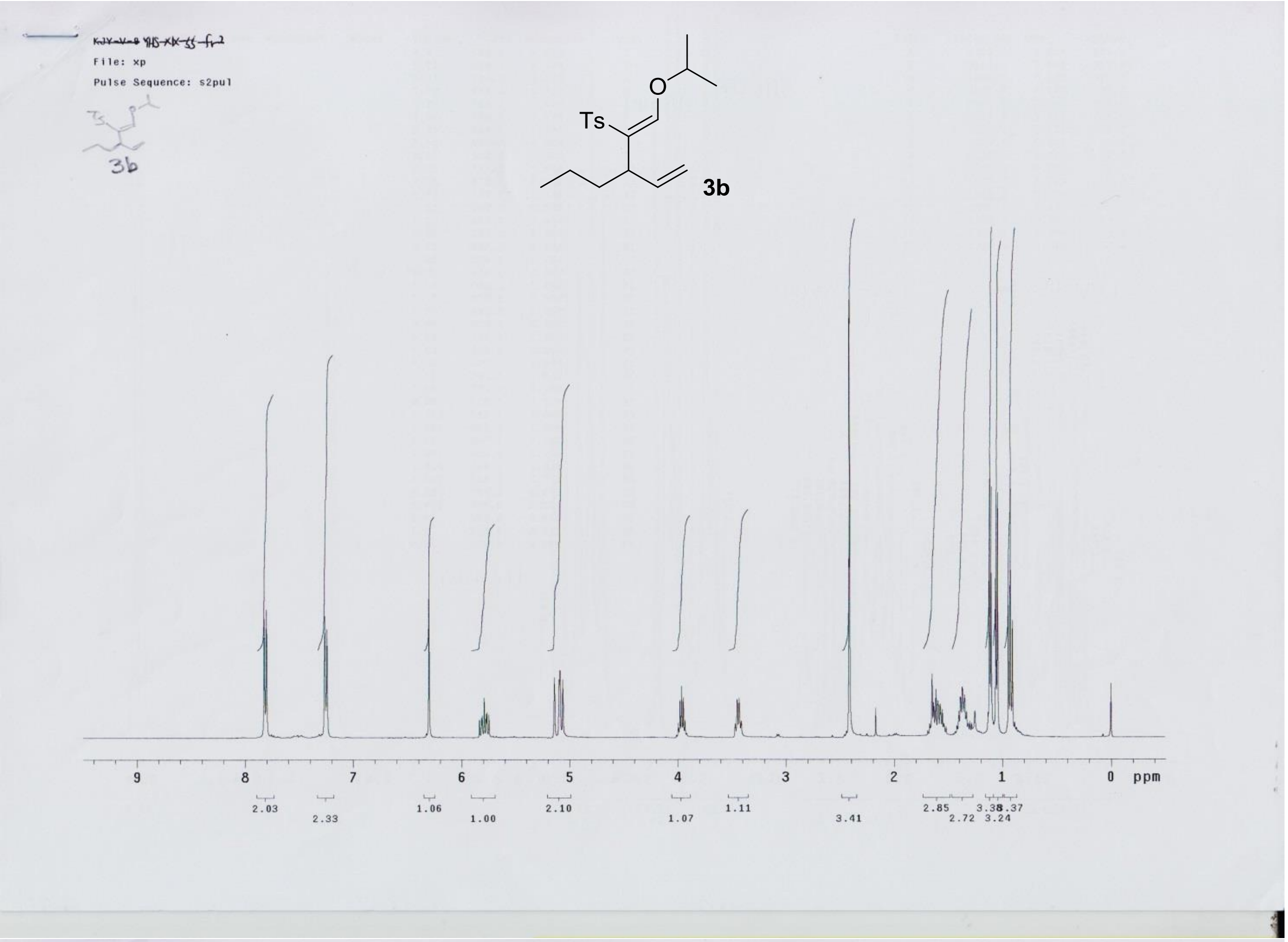
File: xp
Pulse Sequence: s2pul
Ts
O
3b
9 8 7 6 5 4 3 2 1 0 ppm
2.03 2.33 1.06 1.00 2.10 1.07 1.11 3.41 2.85 2.72 3.38 3.24 3.37

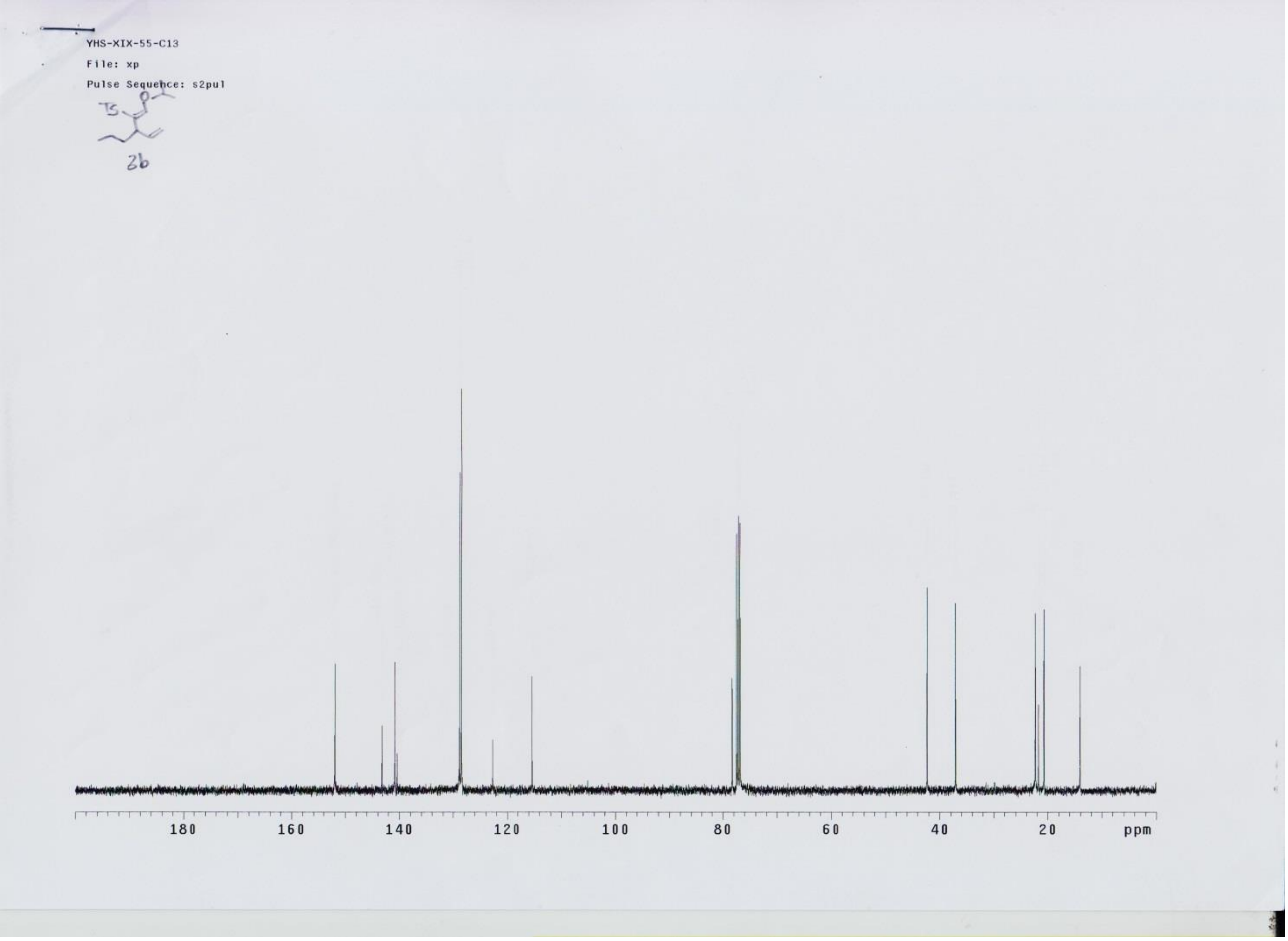
YHS-XIX-55-C13
File: xp
Pulse Sequence: s2pul
180 160 140 120 100 80 60 40 20 ppm

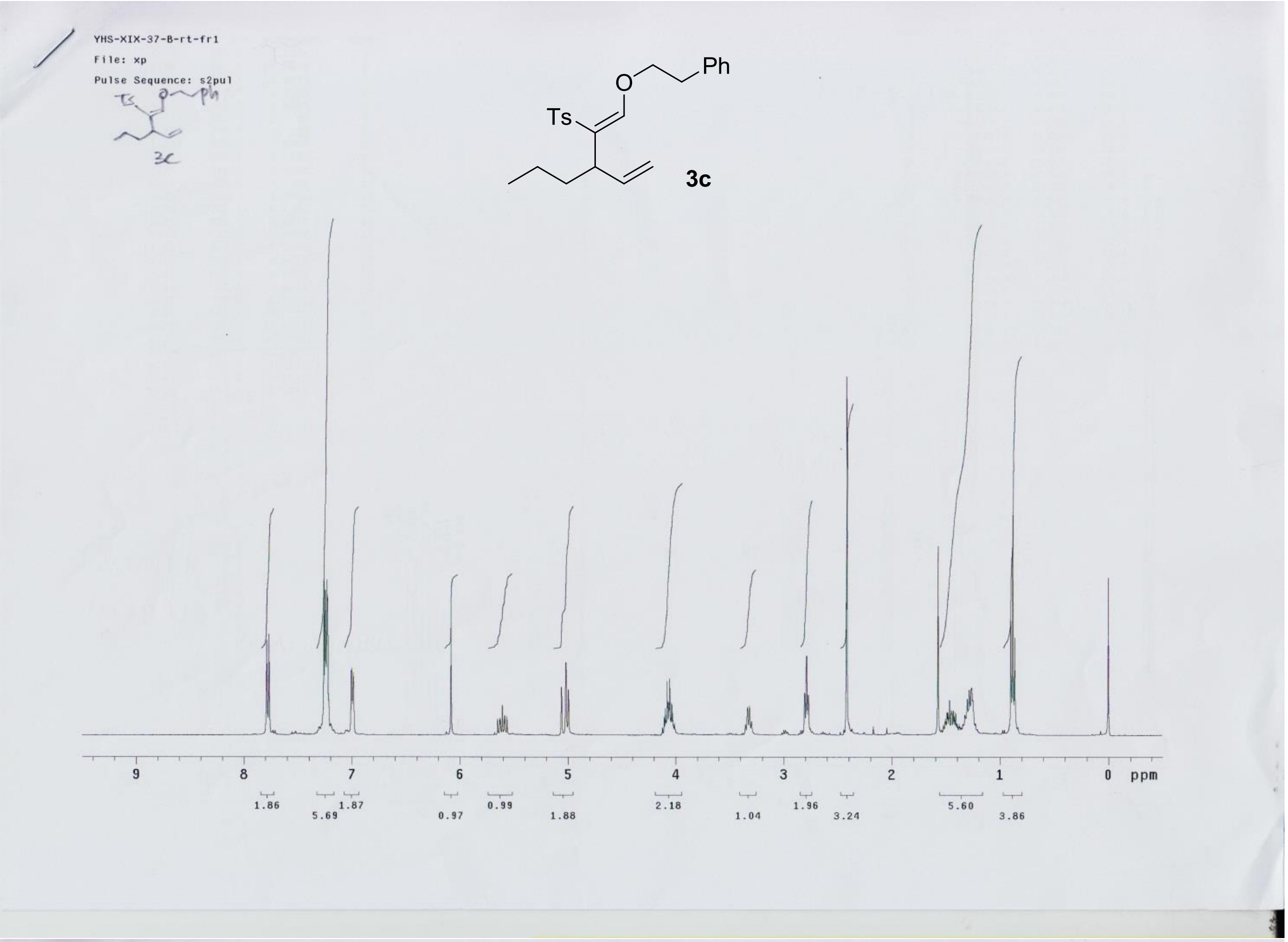
YHS-XIX-37-B-rt-fr1
File: xp
Pulse Sequence: s2pul
Ts
O
Ph
3c
ppm
1.86
5.69
1.87
0.97
0.99
1.88
2.18
1.04
1.96
3.24
5.60
3.86

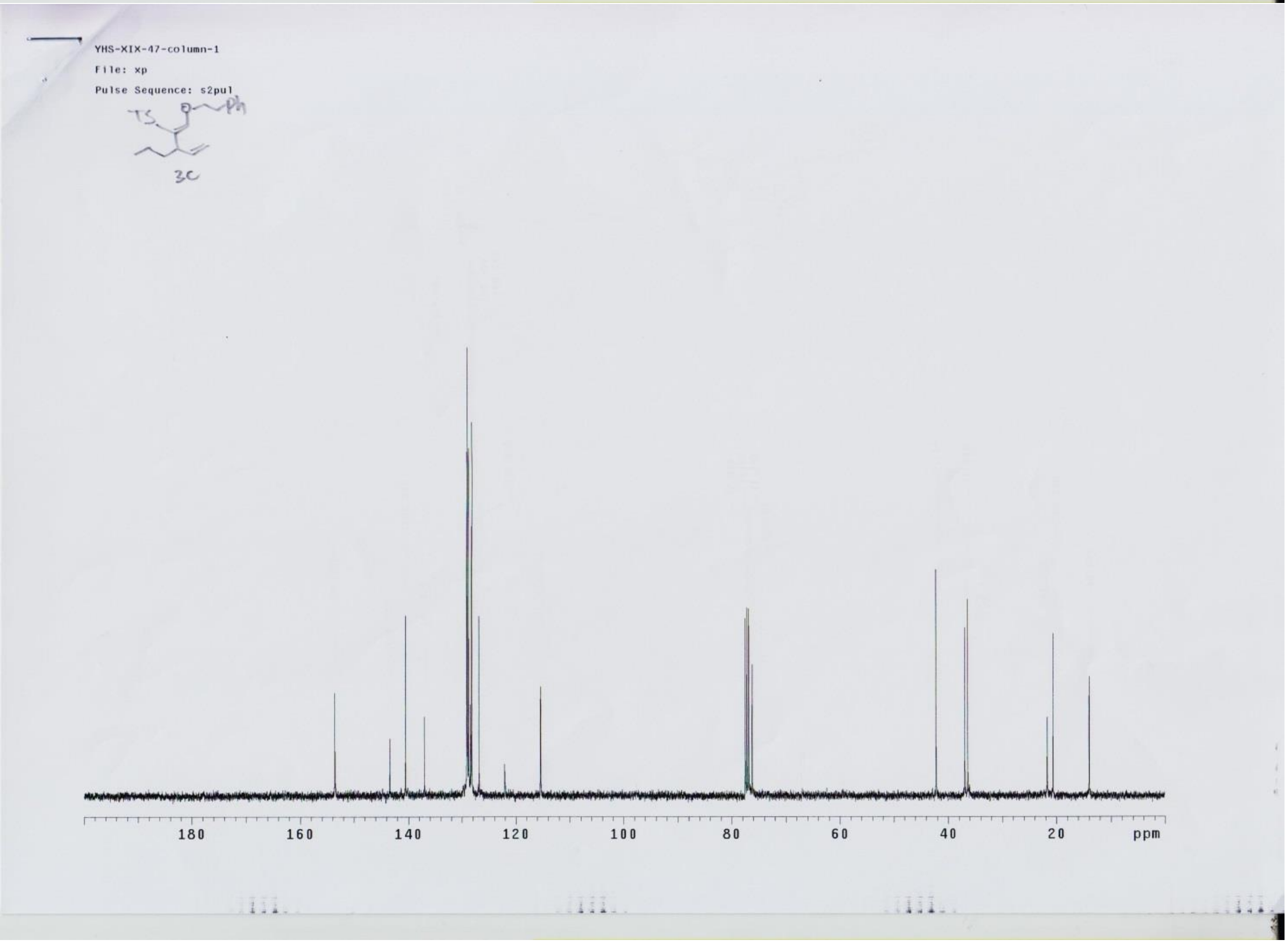
YHS-XIX-47-column-1
File: xp
Pulse Sequence: s2pul
ppm

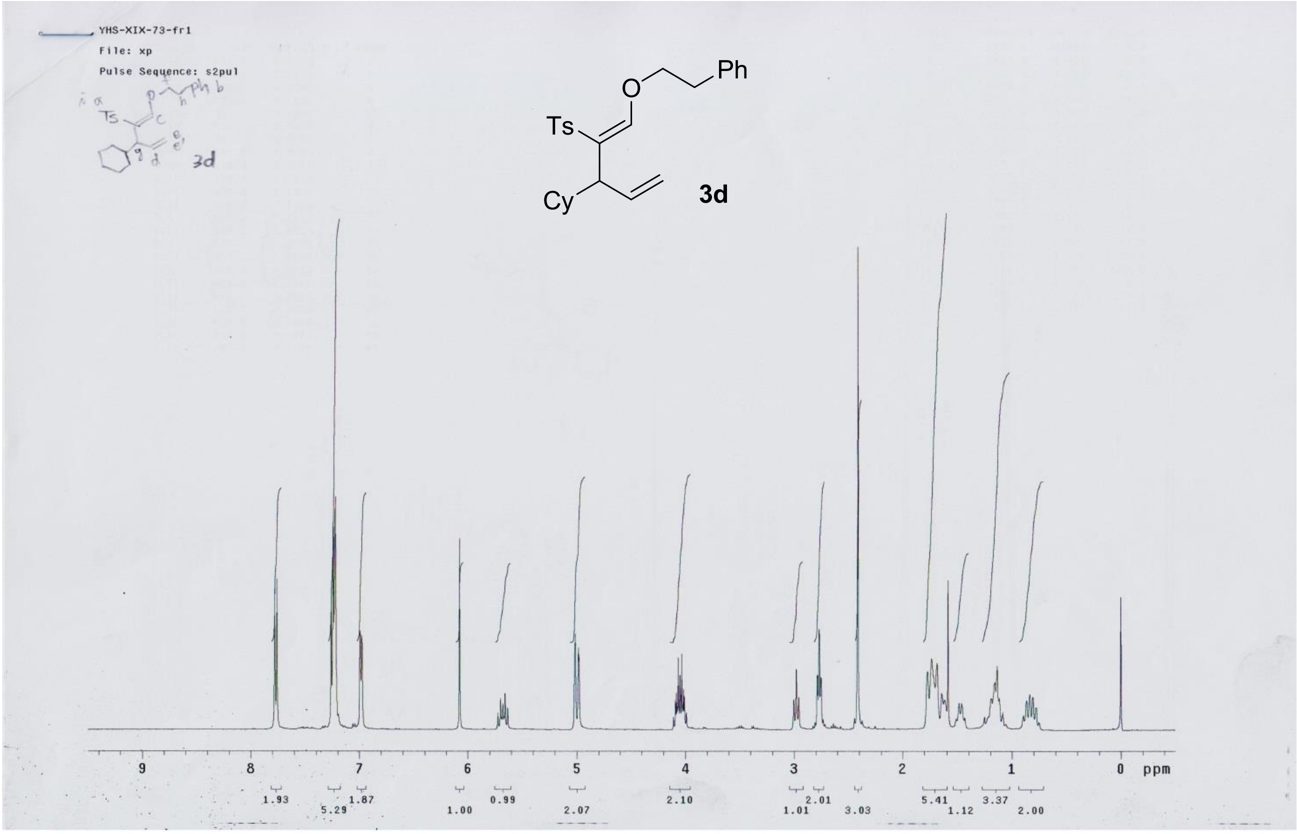
YHS-XIX-73-fr1
File: xp
Pulse Sequence: s2pul
Ts
Ph
O
Cy
3d
9
8
7
6
5
4
3
2
1
0
ppm
1.93
5.29
1.87
1.00
0.99
2.07
2.10
1.01
2.01
3.03
5.41
1.12
3.37
2.00

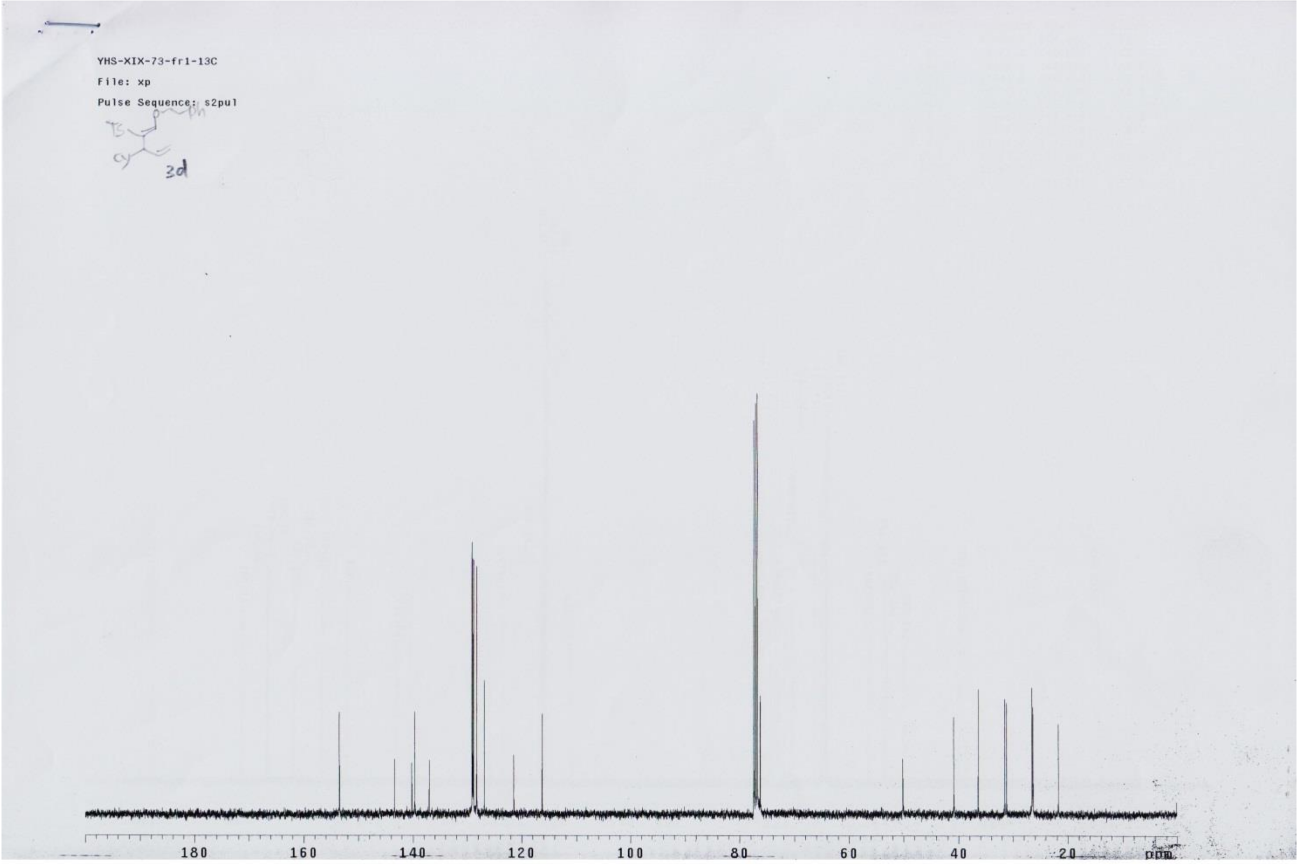
YHS-XIX-73-fr1-13C
File: xp
Pulse Sequence: s2pul
180
160
140
120
100
80
60
40
20
ppm

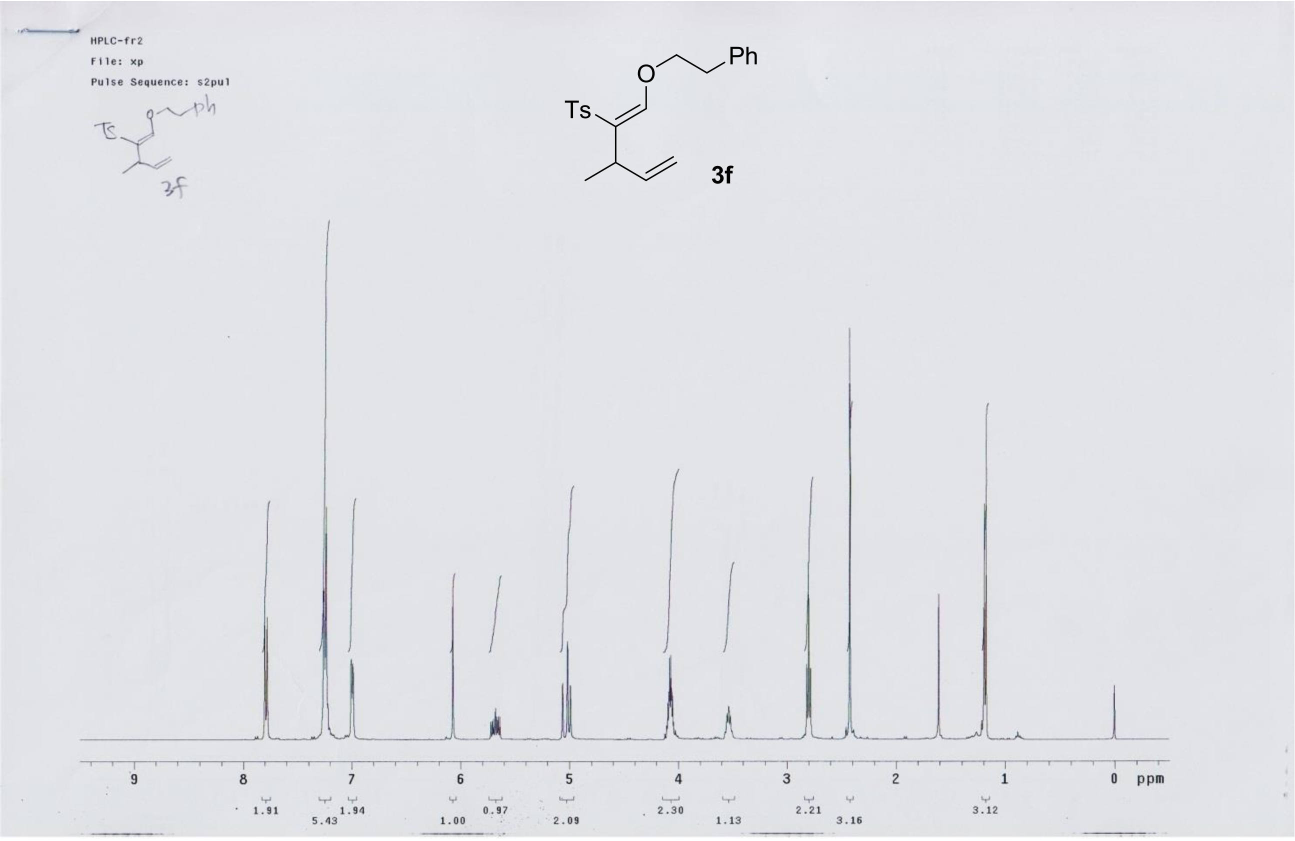
HPLC-fr2
File: xp
Pulse Sequence: s2pul
Ph
O
Ts
3f
9 8 7 6 5 4 3 2 1 0 ppm
1.91 5.43 1.94 1.00 0.97 2.09 2.30 1.13 2.21 3.16 3.12

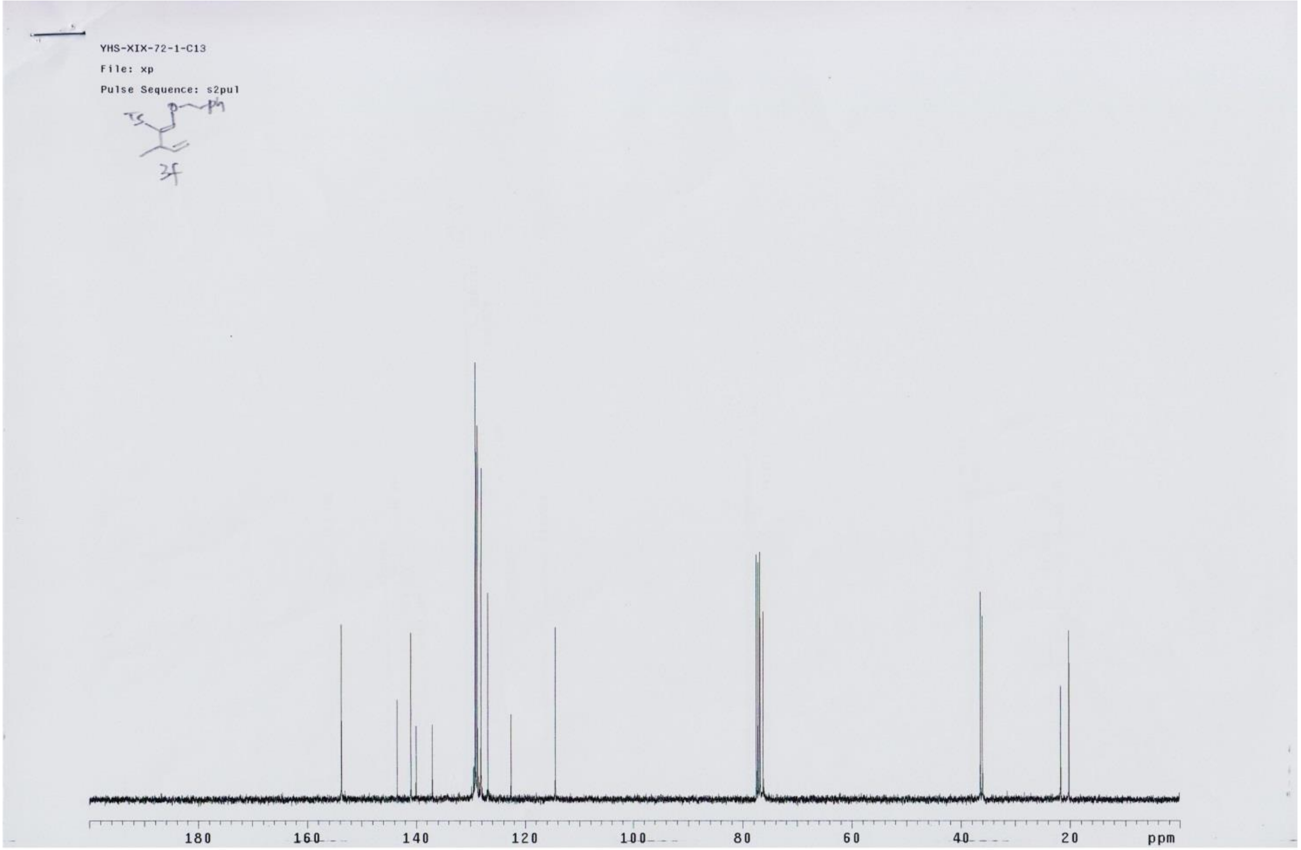
YHS-XIX-72-1-C13
File: xp
Pulse Sequence: s2pul
180 160 140 120 100 80 60 40 20 ppm

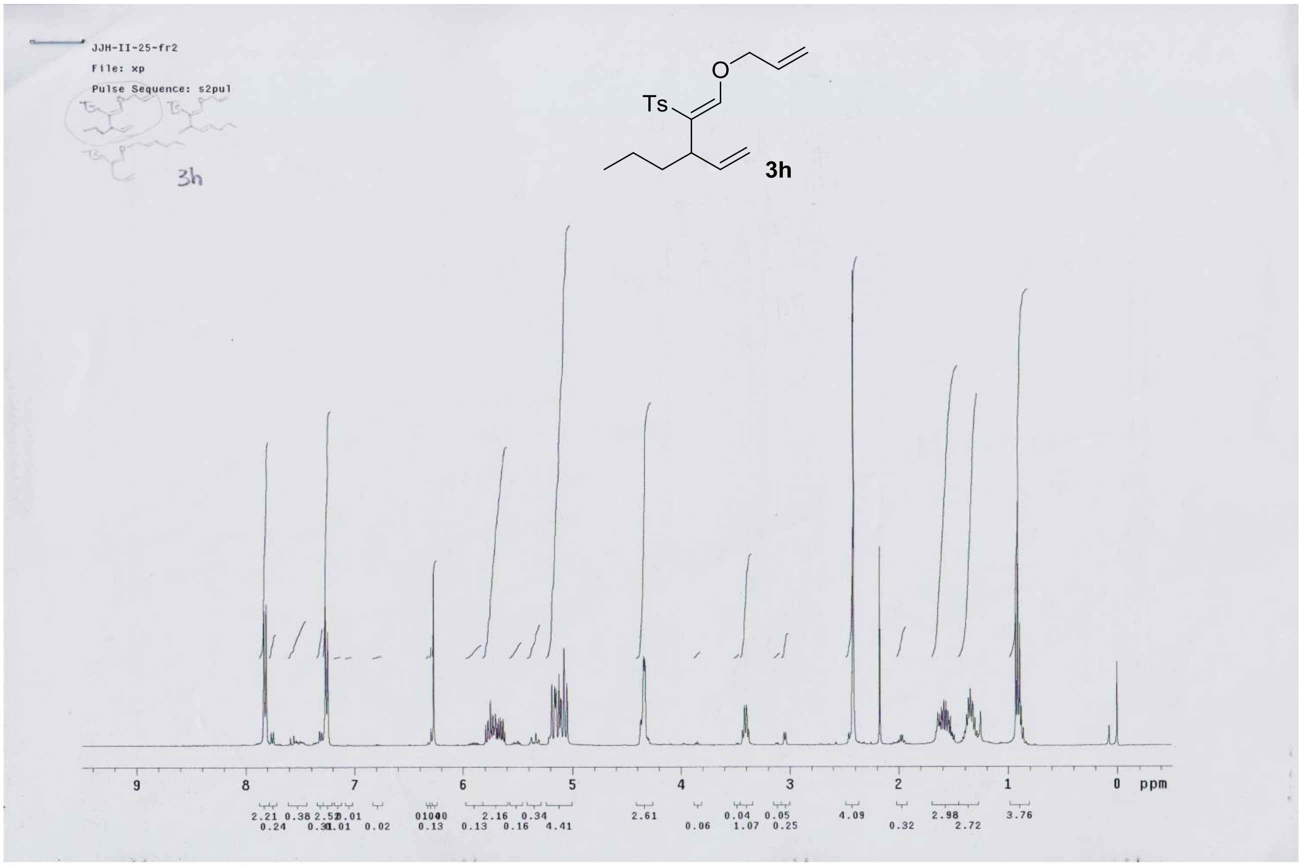
JJH-II-25-fr2
File: xp
Pulse Sequence: s2pul
Ts
O
3h
9 8 7 6 5 4 3 2 1 0 ppm

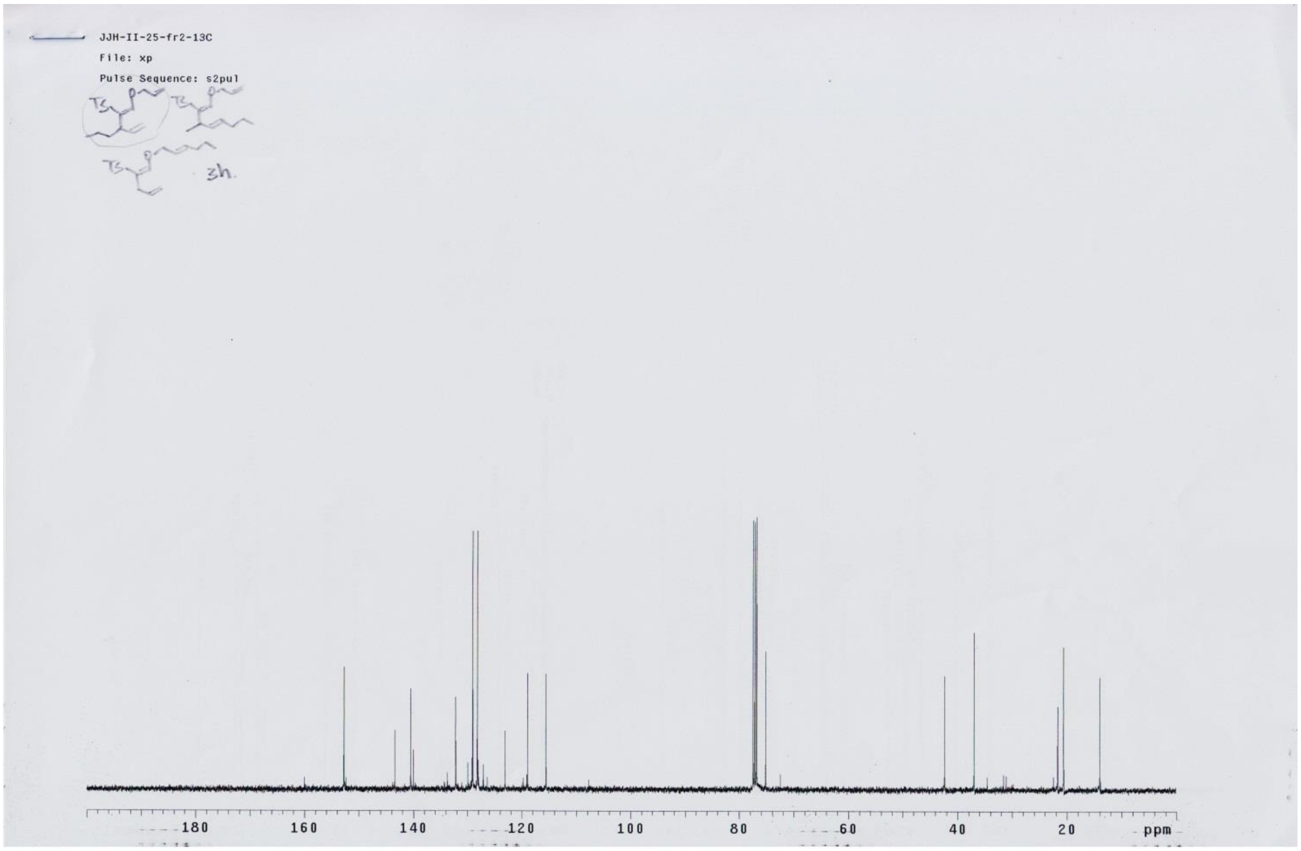
JJH-II-25-fr2-13C
File: xp
Pulse Sequence: s2pul
180 160 140 120 100 80 60 40 20 ppm

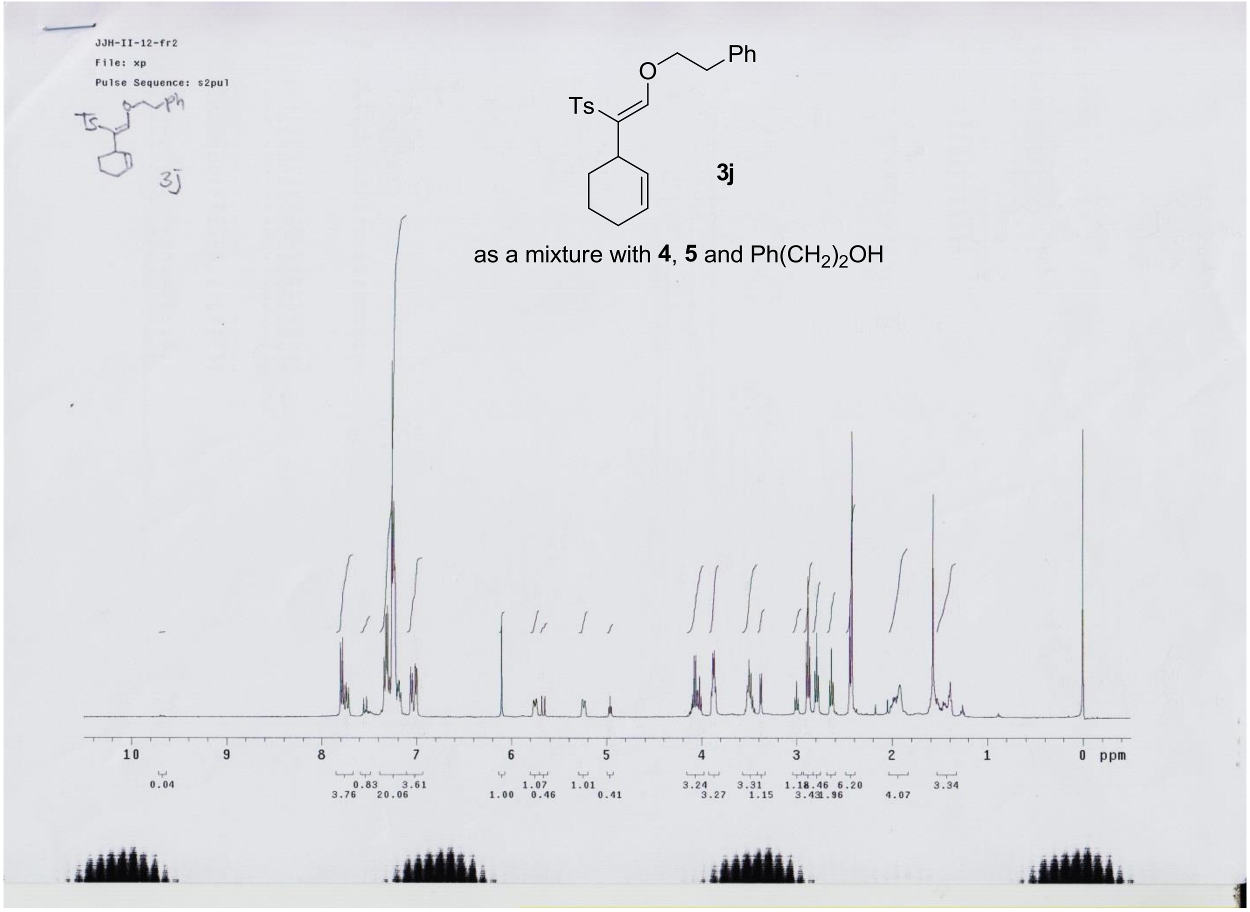
JJH-II-12-fr2
File: xp
Pulse Sequence: s2pul
Ts
O
Ph
3j
as a mixture with 4, 5 and Ph(CH2)2OH
10 9 8 7 6 5 4 3 2 1 0 ppm

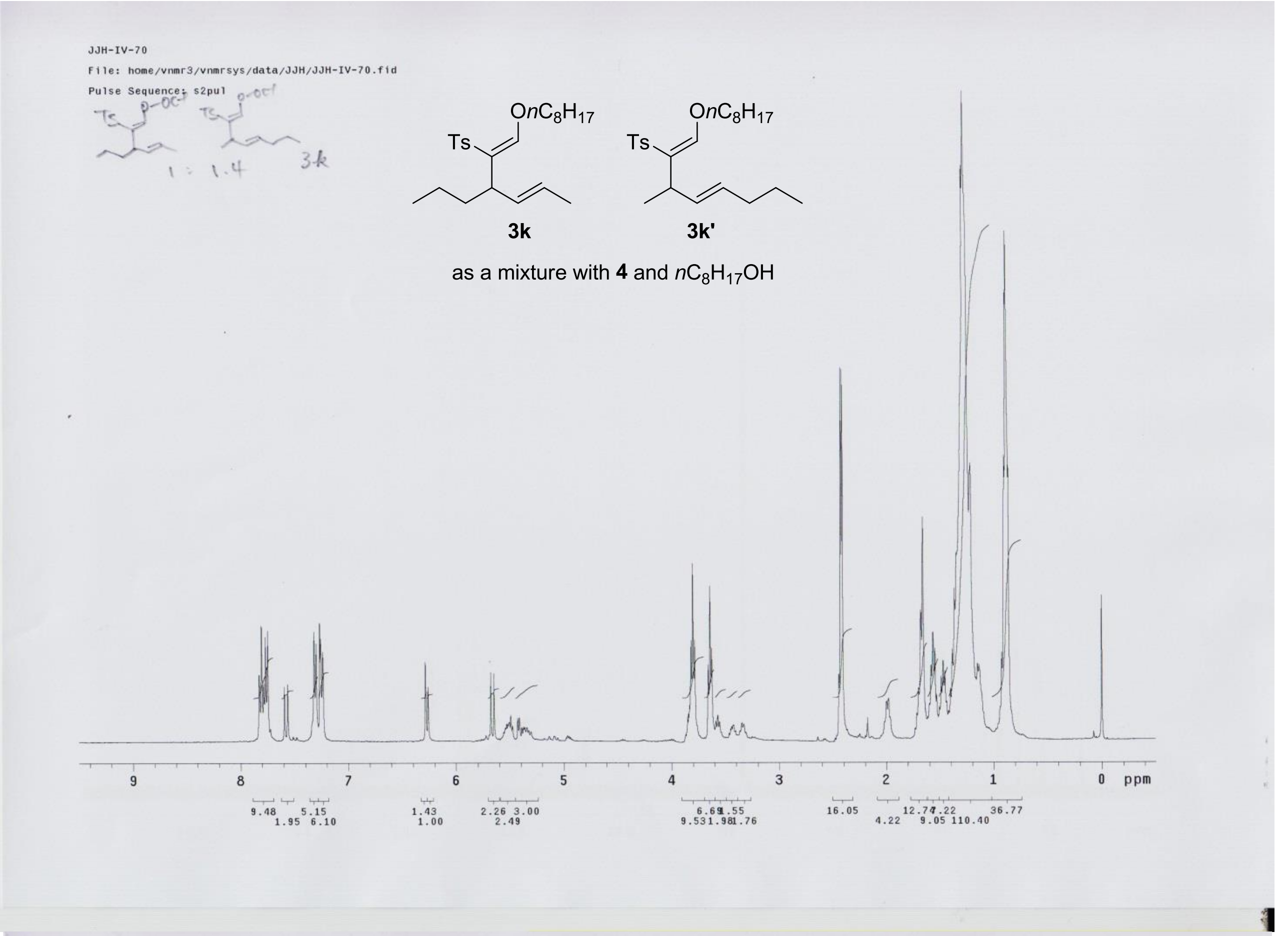
JJH-IV-70
File: home/vnmr3/vnmrsys/data/JJH/JJH-IV-70.fid
Pulse Sequence: s2pul
OnC8H17
Ts
3k
3k'
as a mixture with 4 and nC8H17OH
ppm

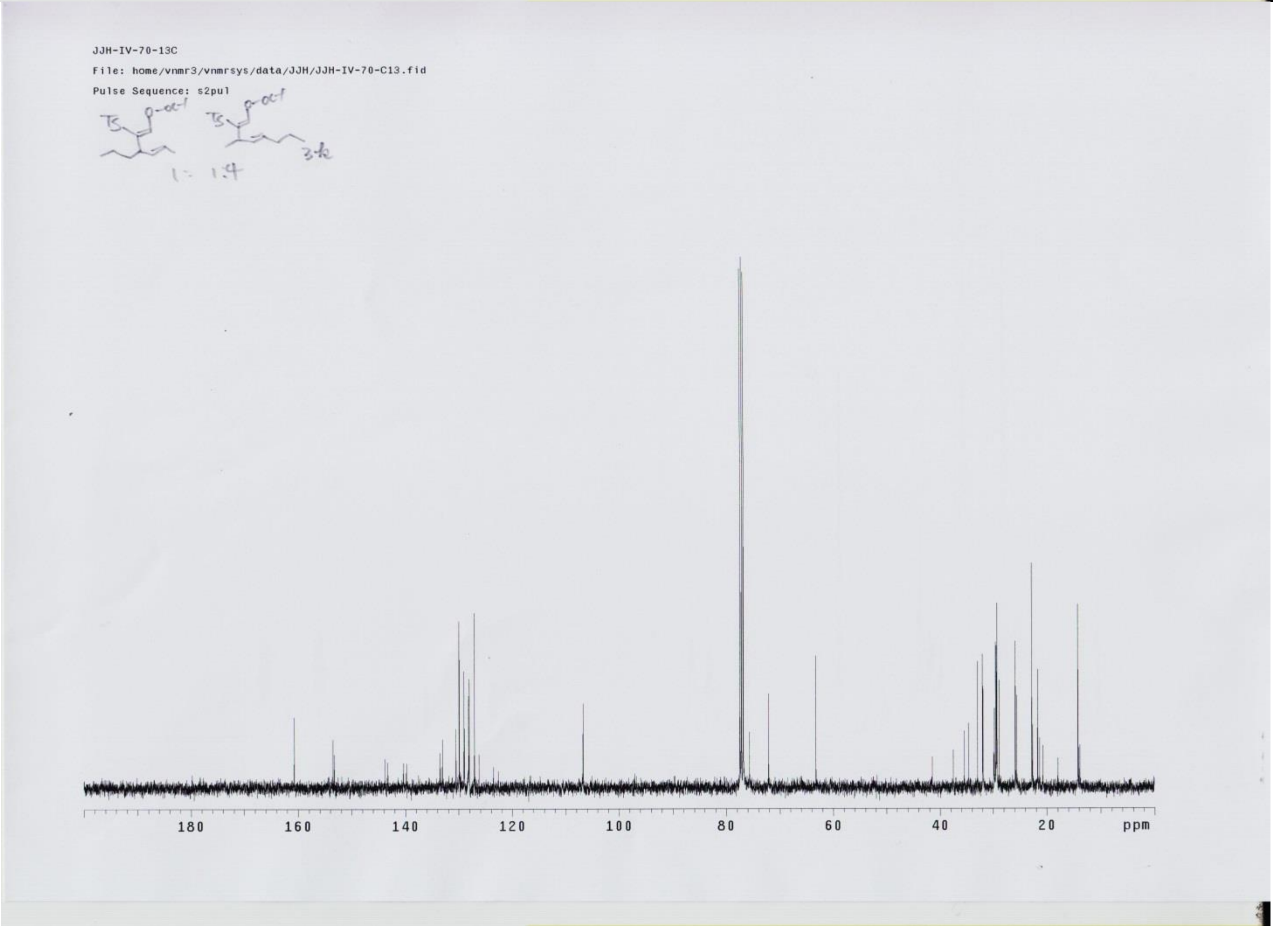
JJH-IV-70-13C
File: home/vnmr3/vnmrsys/data/JJH/JJH-IV-70-C13.fid
Pulse Sequence: s2pul
ppm

Std Carbon experiment
File: home/vnmr3/vnmrsys/data/JJH/JJH-IV-69.fid
Pulse Sequence: s2pul

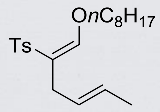

3l

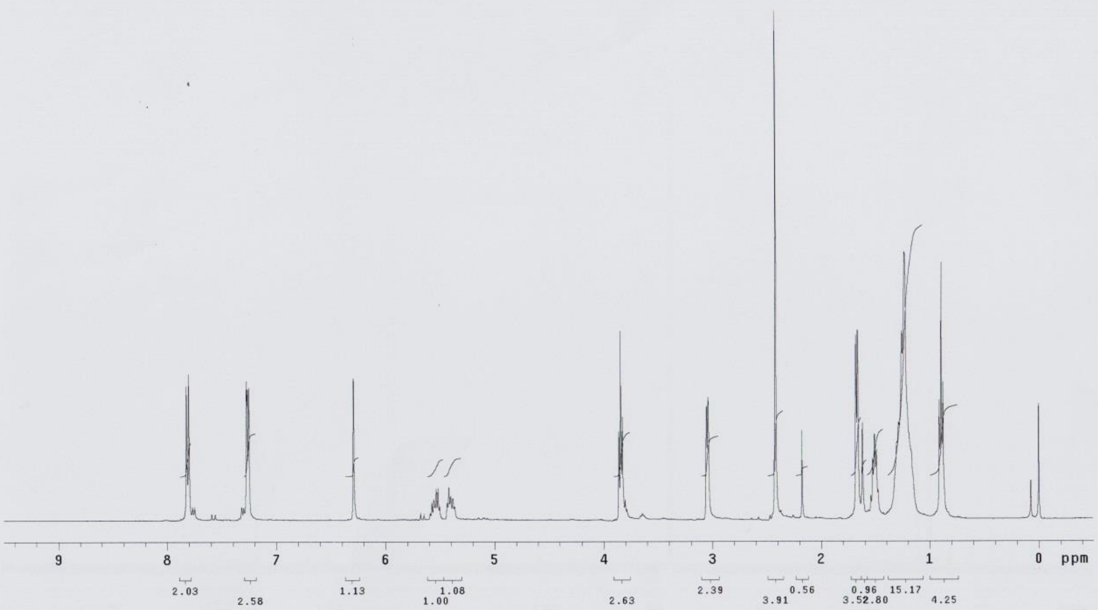

JJH-IV-69-C13
File: home/vnmr3/vnmrsys/data/JJH/JJH-IV-69-C13.fid
Pulse Sequence: s2pul

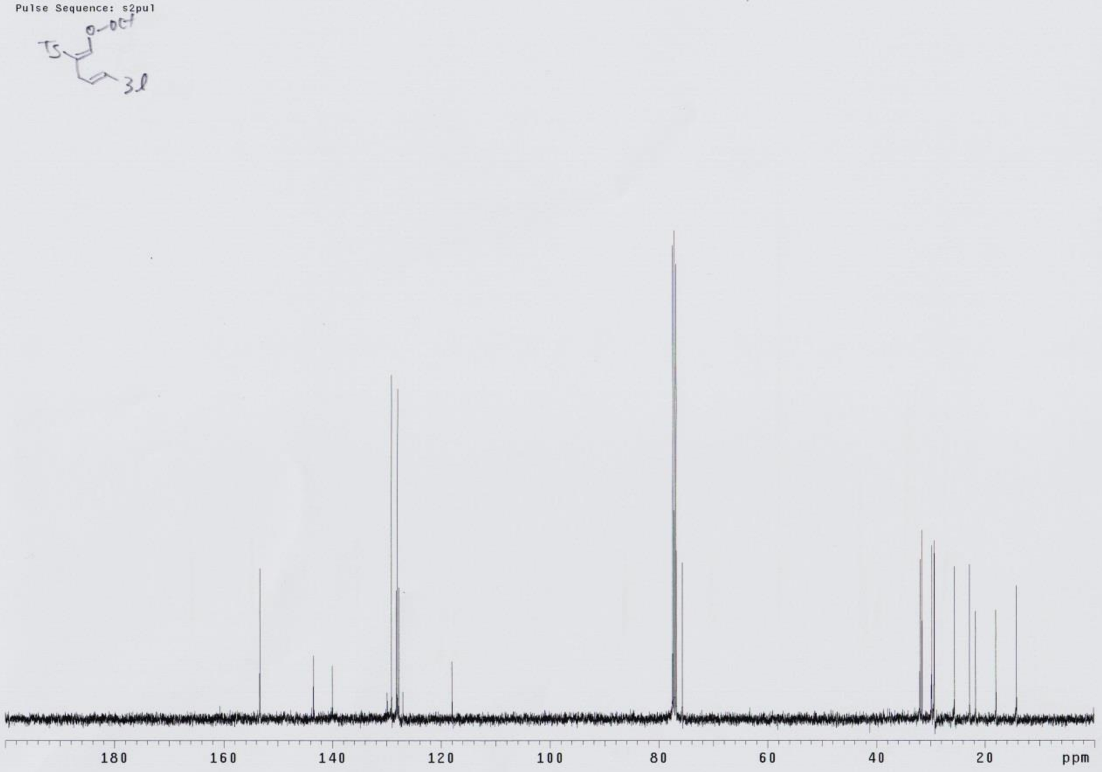

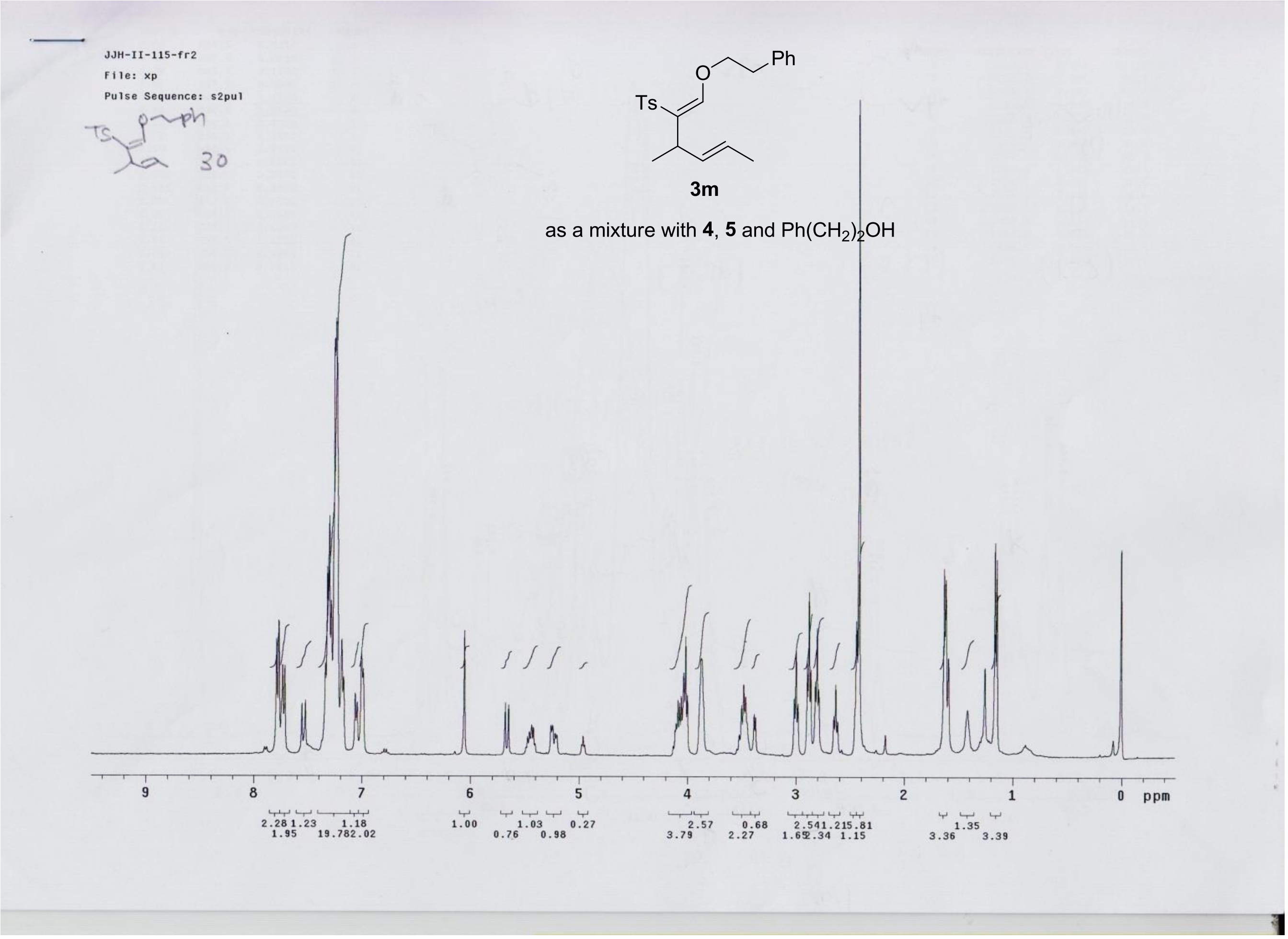
JJH-II-115-fr2
File: xp
Pulse Sequence: s2pul
Ts
O
Ph
3m
as a mixture with 4, 5 and Ph(CH2)2OH
9
8
7
6
5
4
3
2
1
0
ppm
2.28
1.95
1.23
19.78
1.18
2.02
1.00
0.76
1.03
0.98
0.27
3.79
2.57
2.27
0.68
1.65
2.54
2.34
1.21
1.15
5.81
3.36
1.35
3.39

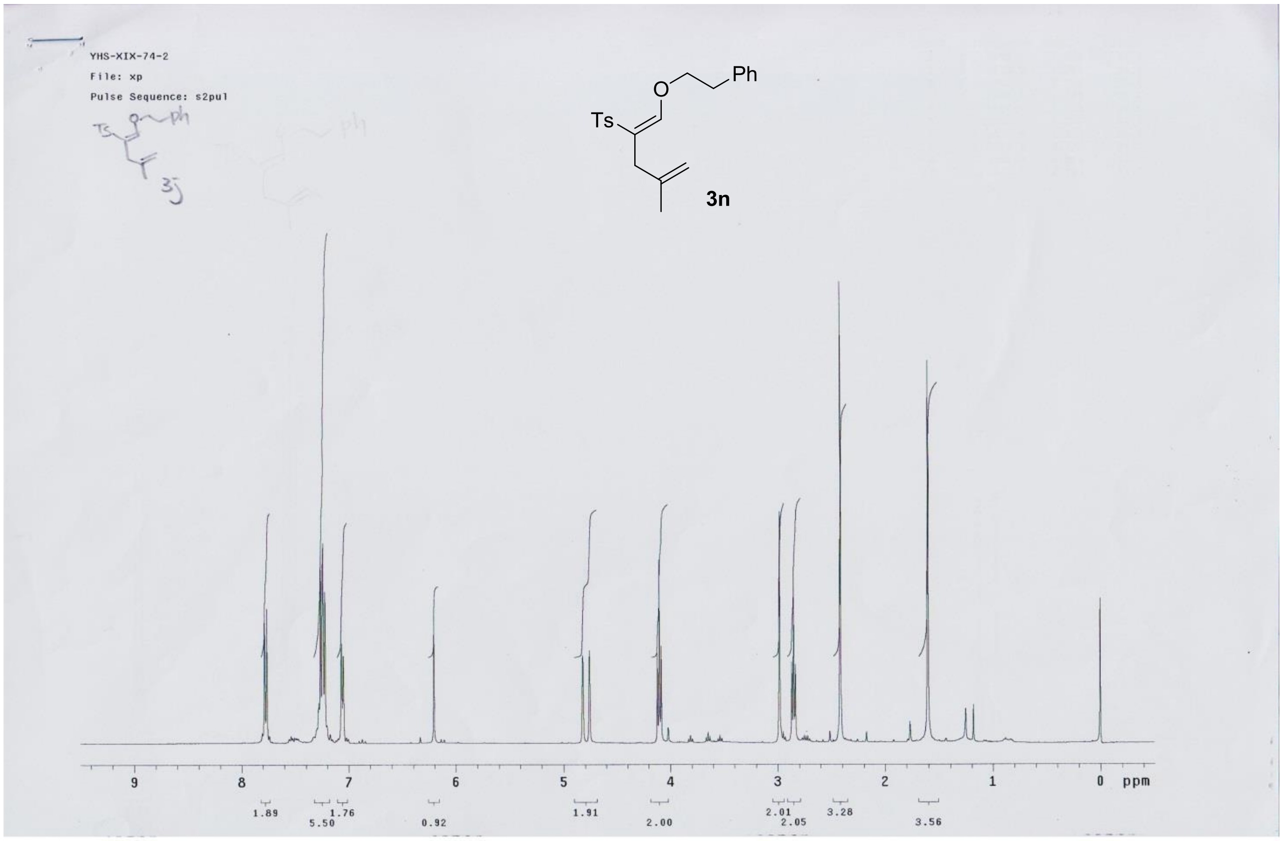
YHS-XIX-74-2
File: xp
Pulse Sequence: s2pul
Ts
O
Ph
3n
9
8
7
6
5
4
3
2
1
0
ppm
1.89
5.50
1.76
0.92
1.91
2.00
2.01
2.05
3.28
3.56

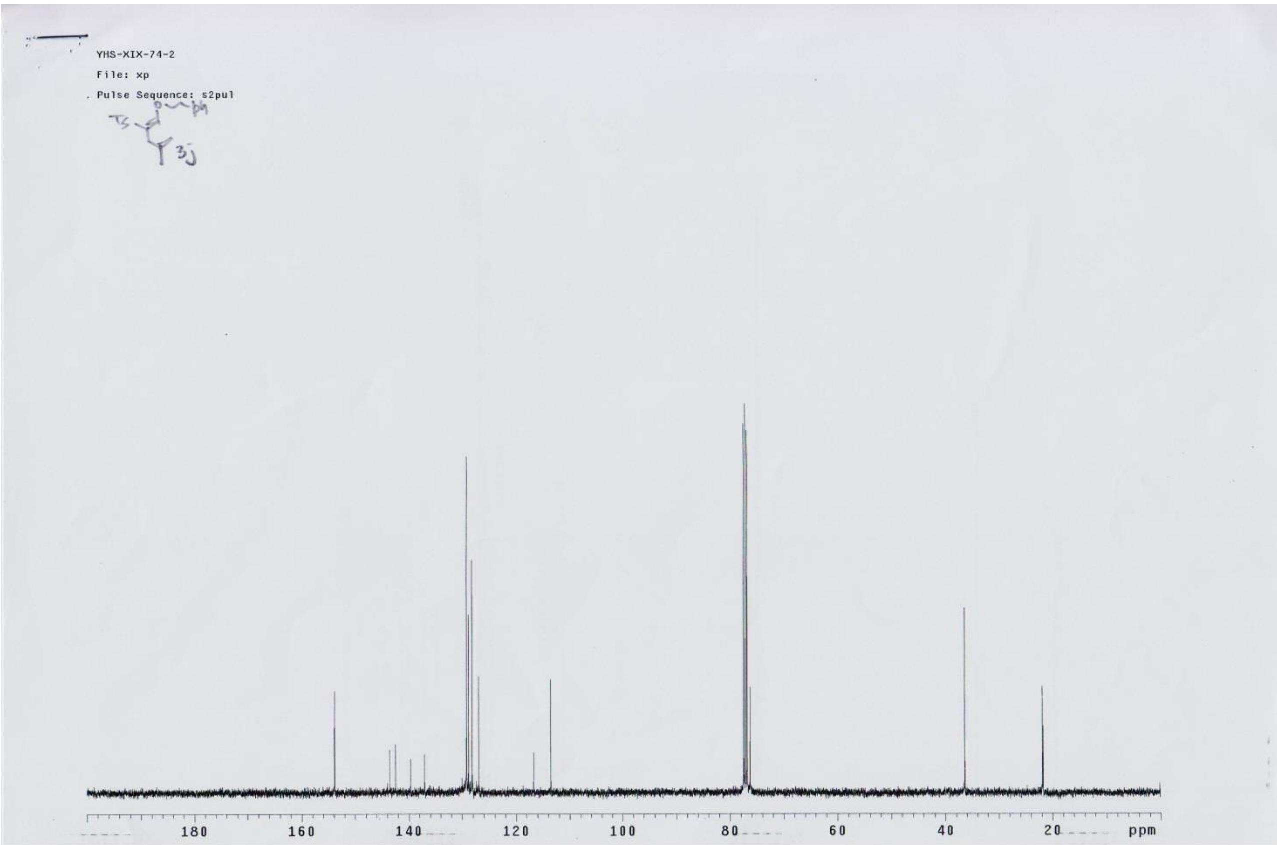
YHS-XIX-74-2
File: xp
Pulse Sequence: s2pul
180
160
140
120
100
80
60
40
20
ppm
